# Targeted fine-tuning of machine-learning interatomic potentials for phonons and phase transitions

**DOI:** 10.1038/s41524-026-02338-w

**Published:** 2026-10-07

**Authors:** Jonas Grandel, Philipp Benner, Janine George

**Affiliations:** 1 Bundesanstalt für Materialforschung und -prüfung (BAM), Berlin, Germany; 2 Institute of Condensed Matter Theory and Solid-State Optics, Friedrich Schiller University, Jena, Germany

**Keywords:** Materials science, Physics

## Abstract

Machine-learning interatomic potentials are widely used as computationally efficient surrogates for density functional theory in atomistic simulations, enabling large-scale and long-time modeling of materials systems. Here, we investigate how different fine-tuning strategies affect the prediction of harmonic phonon band structures, thermal and elastic properties, and the potential-energy surface along unstable phonon modes. We show that substantial improvements can be achieved with very limited additional data, with as few as 10 material-specific training structures. We investigate targeted *L*^2^-SP (*L*^2^-TSP) for this application, combining regularization toward the pre-trained model with selective fine-tuning of the first two representation-building layers, alongside transfer learning and multihead fine-tuning, with an additional comparison to low-rank LoRA for phonon prediction. Across 53 materials, *L*^2^-TSP achieves the most consistent overall performance, reducing phonon errors while also improving predictions of thermodynamic and elastic properties. Importantly, models with similar harmonic phonon accuracy can differ substantially in their prediction of dynamical instabilities and phase-transition behavior. Among the investigated approaches, *L*^2^-TSP shows the best overall agreement in dynamical-stability classification and in recovering the associated DFT reference phases, demonstrating improved generalization beyond the fine-tuning region. We implement *L*^2^-SP and *L*^2^-TSP in *Equitrain*, a model-agnostic software package for training and fine-tuning machine-learning interatomic potentials that can be applied beyond the MACE architecture considered here.

## Introduction

Pre-trained machine-learned interatomic potentials (MLIPs) such as MACE-MP-0b3^1^ are increasingly used to predict ground-state properties and to perform both molecular dynamics and static calculations. Due to recent advances in model architectures and training strategies, modern MLIPs now achieve accuracies approaching those of density functional theory (DFT) for a broad range of atomistic tasks, making them promising alternatives for large-scale and high-throughput calculations. Previous studies have trained MLIPs explicitly for phonon calculations using large datasets^2,3^. In contrast, we investigate whether accurate phonon predictions can be achieved through fine-tuning with only a small number of additional, material-specific training structures. When trained on large and diverse collections of materials spanning a wide range of crystal structures and chemical compositions, pre-trained MLIPs can be viewed as foundation models. In this setting, they provide transferable representations of atomic interactions that generalize across chemical and structural spaces, enabling predictions for materials beyond those explicitly encountered during training^1^.

For certain properties, however, stringent accuracy requirements remain. Phonon calculations, and particularly the prediction of dynamical instabilities, are highly sensitive to small errors in the predicted forces, making them a stringent benchmark for machine-learning interatomic potentials. While certain harmonic-phonon properties, such as heat capacity, already show very good agreement with DFT reference data^4^, accurate prediction of phonon band structures, especially dynamical stability, remains challenging. Moreover, correctly predicting an imaginary phonon mode does not necessarily imply that the underlying potential-energy surface (PES) along the unstable direction is faithfully represented. A model may reproduce the local curvature associated with an instability while still predicting an incorrect anharmonic pathway or final structure.

A variety of fine-tuning strategies^1,5,6^ have been developed to adapt foundation models efficiently to specific systems or target domains. Fine-tuning can reduce systematic errors inherent to foundation models^7^, but naive transfer learning carries the risk of catastrophic forgetting^8^, as parameters optimized on a comparatively small system-specific dataset may drift away from the pre-trained solution. One strategy to restrict this adaptation is layer freezing^5^, in which only selected parts of the network remain trainable. Alternatively, multi-head fine-tuning uses a subset of the foundation model’s original training data as a replay set alongside the new fine-tuning data^1,9^, thereby mitigating catastrophic forgetting at the cost of additional computational effort. Other approaches constrain the parameter updates directly. *L*^2^ starting-point regularization (*L*^2^-SP)^10^ penalizes deviations from the pre-trained parameters, whereas Low-Rank Adaptation (LoRA)^11^ restricts additive parameter updates through a low-rank factorization.

For phonon calculations, fine-tuning additionally raises the question of how to construct an informative material-specific training set. In the finite-displacement approach, as implemented in Phonopy^12,13^, single atoms in supercells are displaced to determine the force constants of the system. Converged calculations generally require sufficiently large supercells and therefore become computationally expensive at the DFT level. Other approaches construct force constants from sets of rattled structures^14^. MLIPs can substantially reduce this computational cost, but their predictive quality for vibrational properties depends on accurately representing the relevant region of the PES.

Since our phonon calculations are based on single-atom-displaced supercells, a naive fine-tuning dataset would consist of such structures. However, because the MACE architecture relies on (semi-)local atomic environments, single-atom-displaced supercells provide limited training information, as most atoms remain close to their equilibrium positions. We therefore construct material-specific fine-tuning datasets designed to sample the region around equilibrium more efficiently. Scaled and deformed rattled structures are relaxed with the MACE-MP-0b3 foundation model (hereafter MP-0b3), and configurations distributed along the relaxation trajectories are selected for DFT calculations. In contrast to approaches that fine-tune a single model across multiple materials^2,3^, we train an individual model for each material.

**Fig. 1:**
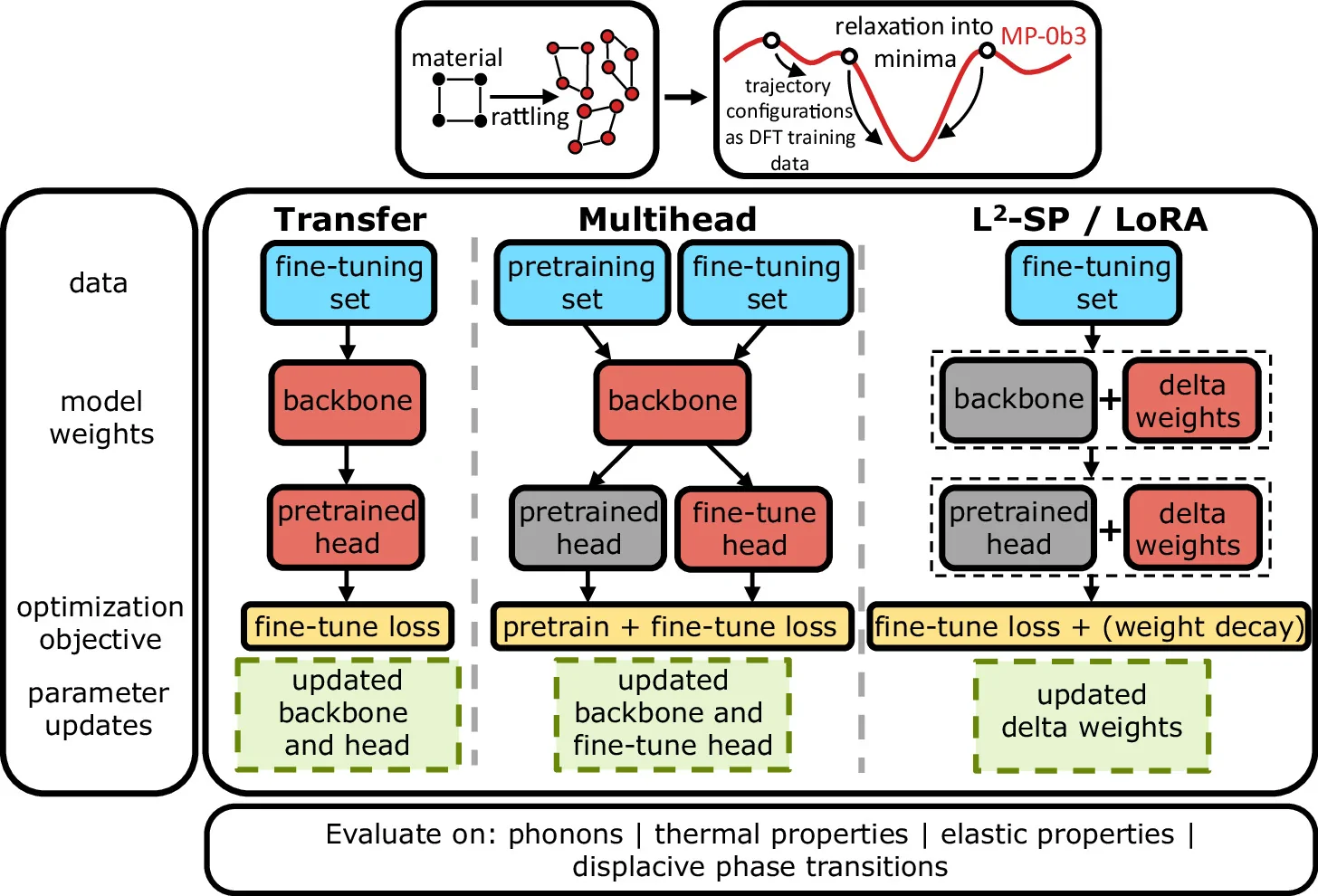
Overview of the concepts and fine-tuning strategies considered in this work. Rattled structures were generated for each material and used to obtain relaxation trajectories with the foundation MP-0b3 model, from which the DFT fine-tuning data set was constructed. The main fine-tuning strategies were evaluated in terms of phonons, thermal and elastic properties, and phase-transition behavior, while LoRA was assessed separately for phonon prediction. Blue boxes indicate the training data, red boxes highlight the model components updated during fine-tuning, gray boxes represent components that remain frozen, yellow boxes denote the optimization objective, and green boxes indicate the resulting parameter updates. Transfer learning (left) updates both the pre-trained backbone and task-specific head using only the fine-tuning objective. Multihead (center) jointly optimizes a shared backbone using both pretraining and fine-tuning losses, while maintaining separate task heads. *L*^2^-SP and LoRA (right) parametrize the model weights as $\omega = \omega_0 + \Delta \omega$. For *L*^2^-SP, a weight decay term is applied to $\Delta \omega$, regularizing deviations from the pre-trained initialization. In *L*^2^-TSP, these updates are additionally restricted to selected layers of the network. LoRA instead constrains the update Δ*ω* through a low-rank parameterization.

Building on these considerations, we combine *L*^2^-SP regularization with selective layer fine-tuning, an approach we refer to as targeted *L*^2^-SP (*L*^2^-TSP). By regularizing deviations from the pre-trained solution while restricting adaptation to selected parts of the network, *L*^2^-TSP provides a constrained approach to material-specific adaptation. We implement *L*^2^-SP and *L*^2^-TSP in the fine-tuning software package *Equitrain*^15^, which is not restricted to the MACE architecture considered here and can be applied to other MLIP models. Figure 1 summarizes the overall workflow, from the generation of material-specific fine-tuning data through model adaptation to the evaluation of phonon, thermal, elastic, and phase-transition properties.

In this work, we systematically compare transfer learning, multi-head fine-tuning, and *L*^2^-TSP across 53 materials. We first assess the data efficiency of the different strategies and investigate how the size of the training structures affects phonon accuracy. We then go beyond global phonon-error metrics, which provide only a statistical measure of agreement and do not directly reveal how errors propagate into physical observables or whether improvements in harmonic accuracy translate into faithful predictions away from the near-equilibrium training configurations. Specifically, we evaluate thermal and elastic properties, dynamical instabilities, and the PES along unstable phonon modes. In particular, we test whether models with comparable harmonic phonon accuracy and dynamical-stability predictions also retain sufficient fidelity of the PES to recover the associated relaxed phases when displacements along unstable phonon modes are applied.

LoRA is evaluated separately as a methodological comparison because it shares with *L*^2^-TSP the use of additive updates to a frozen pre-trained model, but constrains these updates through a low-rank parameterization rather than through starting-point regularization and targeted layer selection. This comparison allows us to assess whether low-rank adaptation provides comparable harmonic phonon accuracy to the targeted *L*^2^-SP configuration. We perform this comparison across several LoRA ranks in the Supplementary Information and use the outcome to determine which fine-tuning strategies are carried forward to the more extensive property-level analysis.

## Results and discussion

### Fine-tuning strategies

Fine-tuning is used to adapt pre-trained foundation MLIPs to new materials systems or target properties using limited additional data. In this study, we compare transfer learning, multi-head fine-tuning, targeted *L*^2^-SP (*L*^2^-TSP), and LoRA. Transfer learning and multi-head fine-tuning are performed using the MACE package^9,16^. In transfer learning, the pre-trained model parameters are directly optimized on the fine-tuning data, whereas multi-head fine-tuning additionally replays data from the original training set to mitigate catastrophic forgetting. This additional replay data, however, increases the computational cost of training.

*L*^2^-TSP follows a different strategy by combining *L*^2^-SP regularization with selective layer fine-tuning. *L*^2^-SP penalizes deviations from the pre-trained parameters, while the targeted fine-tuning restricts adaptation to a selected subset of the network. Selective or “surgical” fine-tuning has previously been shown to improve adaptation under distribution shifts, with the optimal subset of layers depending on the nature of the shift^17^. In particular, fine-tuning early layers can be advantageous when adaptation primarily requires changes to low-level representations. In our case, we restrict adaptation to the first two layers of the MACE model, which yields the best performance among the layer subsets considered for *L*^2^-TSP (see Supplementary Information Section “Effect of layer selection in transfer learning and L2-SP fine-tuning” and SI Table S9). We refer to this strategy as targeted *L*^2^-SP (*L*^2^-TSP). *L*^2^-SP and *L*^2^-TSP are implemented in *Equitrain*^15^.

For comparison, we additionally evaluate LoRA^11^ as implemented in MACE. LoRA keeps the corresponding pre-trained weights fixed but constrains their additive updates through a low-rank factorization. Thus, *L*^2^-TSP and LoRA provide a particularly informative comparison: both adapt a frozen pre-trained model through additive updates, but impose different constraints on the adaptation. While *L*^2^-TSP combines regularization toward the pre-trained solution with targeted parameter selection, LoRA restricts the dimensionality of the update through its low-rank parameterization. We therefore evaluate LoRA separately to isolate the effect of low-rank adaptation from the regularization and layer-selection mechanisms employed in *L*^2^-TSP (see SI Section “Comparison of *L*^2^-TSP with LoRA” and SI Table S8). Further details on *L*^2^-SP, *L*^2^-TSP and LoRA are provided in the Methods Section.

### Data efficiency of fine-tuning strategies

**Fig. 2:**
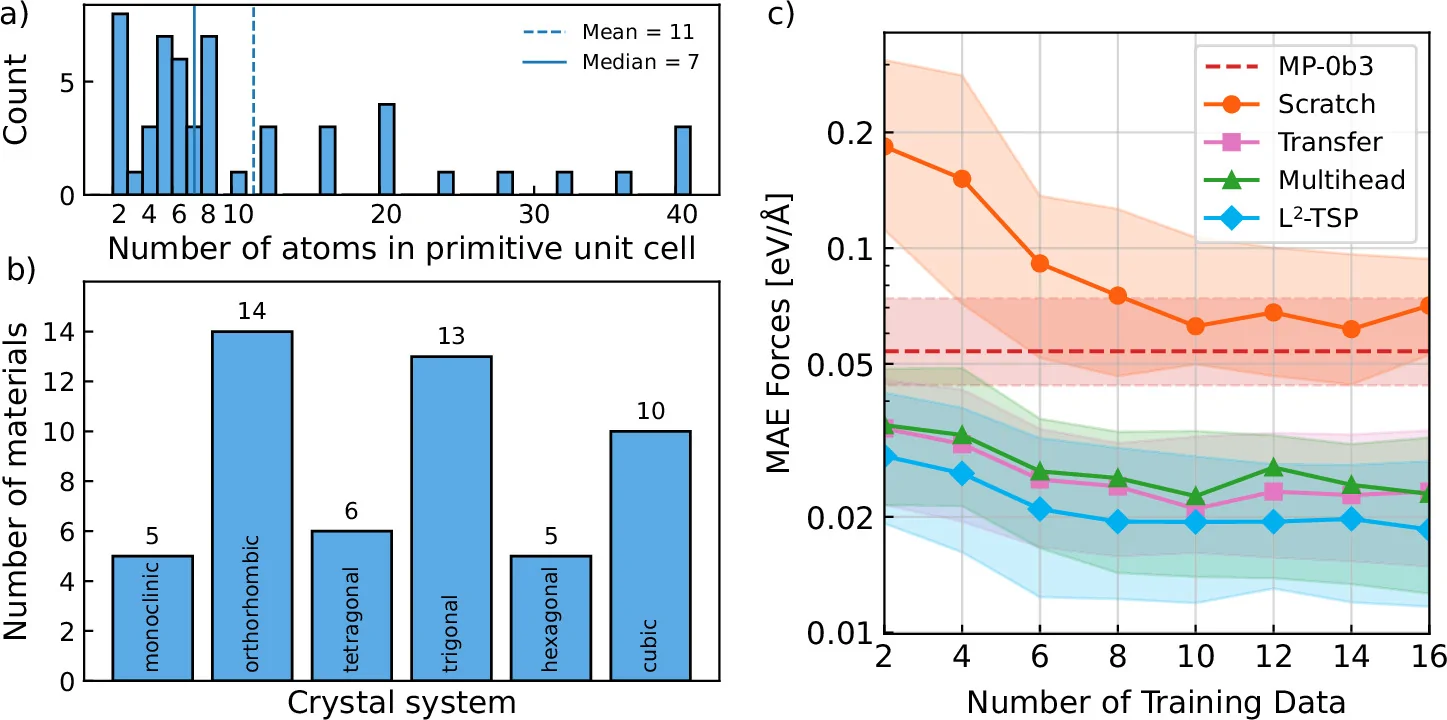
Dataset composition and force-error convergence characterize the data efficiency of fine-tuning. **a** Number of atoms per primitive unit cell and **b** distribution of crystal systems in the dataset, which contains a total of 53 materials. **c** Median force MAEs across all 53 material-specific models within the IQR, as shown in the shaded area in the background. Models trained from scratch (orange circles), transfer learning (violet squares), multi-head fine-tuning (green triangles), and *L*^2^-TSP (blue diamonds) are shown. Note that the y-axis is logarithmic, and lines are drawn between data points for improved readability. The performance of the foundation model, MP-0b3, on the validation data is represented by a red dashed horizontal line for comparison.

For a rigorous evaluation, we calculate phonon band structures for 53 materials. Figure 2a) and b) summarizes the distribution of crystal systems and the number of atoms per unit cell in the dataset. The chosen materials cover all crystal systems except the triclinic one and include not only binary or ternary compounds but also structurally more complex systems with up to 40 atoms per unit cell. The dataset is dominated by phase-change materials and chalcogenide-based semiconductors reflecting their relevance for thermoelectric applications, in which phonon properties play a central role^18^. Most materials contain tellurium, antimony, or selenium, and the element heatmap is shown in SI Fig. S1. All materials were selected from the Materials Project database^19,20^.

In a first step, we determine how much additional, system-specific training data is required for fine-tuning. Because our main interest lies in harmonic phonon calculations, we focus on fine-tuning the models using configurations sampled in the vicinity of the equilibrium structure so that a harmonic potential can be approximated well by the MLIP. The training data comprise rattled structures and selected configurations sampled along relaxation trajectories obtained using the MP-0b3 foundation model. Detailed information on the data generation protocol is provided in the Methods section. To quantify data efficiency, we systematically increase the number of training configurations and fine-tune an individual model per material and strategy. We examine the energy, force, and stress prediction errors for the individual validation sets of each model across different fine-tuning strategies. Because phonon properties are particularly sensitive to force accuracy, we focus here on force mean absolute errors (MAE). The corresponding energy and stress results are consistent with the same overall trend across all training structure sizes and are listed in SI Fig. S2. Figure 2c) compares the force MAE achieved by different fine-tuning strategies when trained on large-supercell datasets. For transfer learning, multi-head fine-tuning, and *L*^2^-TSP, the median force MAE decreases and converges fast as the training set grows. The shaded region shows the interquartile range (IQR), highlighting the variation in fine-tuning performance across materials.

Relative to the MP-0b3 foundation model, transfer learning, multi-head fine-tuning, and *L*^2^-TSP all achieve a clear improvement in force MAE. Models trained from scratch perform worst overall, highlighting the advantage of pre-trained models when training data are limited. Among the fine-tuning approaches, *L*^2^-TSP achieves the lowest force MAE overall, followed by transfer learning and multi-head fine-tuning. The comparatively slightly inferior performance of the latter may be related to the additional, non-system-specific replay dataset used during fine-tuning. Layer freezing can be applied within both transfer learning and *L*^2^-SP and is therefore regarded here as a mechanism for selecting the trainable part of the network rather than as a separate fine-tuning strategy. Its effect on both approaches is analyzed separately in SI Section “Effect of layer selection in transfer learning and *L*^2^-SP fine-tuning”, where the best layer-selected transfer-learning configuration can match *L*^2^-TSP in harmonic phonon accuracy, but remains below *L*^2^-TSP in the more stringent phase-recovery analysis (see SI Table S9). For the subsequent main-text property-level analysis, we therefore focus on transfer learning (without layer freezing), multi-head fine-tuning, *L*^2^-TSP, and from-scratch models trained on 10 configurations per material and compare them against the foundation MP-0b3 model.

### Effect of training structure size on phonon accuracy

We next assess how the size of the training structures affects phonon accuracy. For this, we test primitive unit cells, small cubic supercells with a maximal edge length of 10 Å, and large cubic supercells with a maximal edge length of 15 Å. To evaluate the quality of the phonon band structure calculations within the different fine-tuning strategies, we measure the MAE between the DFT-calculated and the ML-calculated phonon frequencies in the whole Brillouin zone. The results are shown in Table 1. In the case of the primitive and small supercell from-scratch models, phonons could not be calculated for two materials because the structures relaxed into nonphysical configurations. All fine-tuning techniques achieve significant improvements in predicting phonon band structures. Two main trends can be identified in the data. First, we observe a decrease in overall MAE from small to large training structures. This behavior is expected because the number of atoms, and therefore the number of diverse local environments that the model encounters during training, increases in larger structures. Additionally, larger supercells capture long-range force information, which is necessary for accurate phonon calculations.

**Table 1:** Median phonon mean absolute errors (MAE) and interquartile ranges (IQR) in THz of all 53 material models within different fine-tuning strategies and different training structure sizes

	Scratch	Transfer	Multihead	*L*^2^-TSP
primitive	0.39 (0.50)	0.12 (0.07)	0.18 (0.16)	0.12 (0.12)
small supercell	0.25 (0.39)	0.09 (0.06)	0.12 (0.06)	0.07 (0.06)
large supercell	0.21 (0.14)	0.06 (0.05)	0.07 (0.06)	**0.05 (0.05)**

The IQRs are given in parentheses. The lowest MAE and IQR across all fine-tuning strategies and training structure sizes is shown in bold.

Secondly, *L*^2^-TSP is the most successful strategy and achieves the lowest median phonon MAE (shown in bold). For comparison, the foundation MP-0b3 model shows a median MAE (IQR) of 0.27 THz (0.18 THz), which is more than five times higher than the *L*^2^-TSP approach trained with large supercells. Additional comparisons with foundation models trained on larger data sets can be found in SI Section “Foundation model comparison” and SI Table S1.

Given the significant performance advantage of large-supercell training, all subsequent comparisons are limited to models trained on large supercells. We start by comparing the maximum and mean phonon frequencies, and the density of states reported in Table 2. Negative signs in the table indicate that the ML approaches are underestimating the reference results. Overall, the fine-tuning approaches show smaller or comparable errors to the foundation MP-0b3 model, while the from-scratch models perform worst, as expected, across all three quantities. For the prediction of ${\omega }_{\max }$, the *L*^2^-TSP approach performs almost twice as well as the transfer learning approach and three times as well as the multi-head strategy. A main reason for the poor MAE of the foundation MP-0b3 model is a systematic underprediction of maximum phonon frequencies due to systematic softening in foundational MLIPs^7^. Forces are underestimated, resulting in a smaller ${\omega }_{\max }$ and thus a poorer MAE. By fine-tuning, the softening effect is compensated to varying degrees, depending on the strategy. A similar underestimation of phonon frequencies was observed in our earlier study, where phonon properties were computed directly using the MP-0b3 foundation model^1^. Parts of the benchmark data overlap with the dataset used in that study. For $\omega_{{\rm{mean}}}$, all models perform equally well. This suggests that, to calculate properties that depend only on averaged frequencies, the foundation model is already sufficient. Compared to the foundation model, all fine-tuned models show improved predictions of the phonon density of states (DOS), with the *L*^2^-TSP approach achieving the best performance.

**Table 2:** Median error and IQR of maximal phonon frequencies (${\omega }_{\max }$), mean phonon frequencies ($\omega_{{\rm{mean}}}$) and density of states (ΔDOS) for large-supercell training data

	MP-0b3	Scratch	Multihead	Transfer	*L*^2^-TSP
$\Delta {\omega }_{\max }\,[ \% ]$	−7.0 (9.0)	−7.9 (14.4)	−3.1 (4.5)	−1.6 (4.2)	**−0.9 (3.8)**
$\Delta {\omega }_{{\rm{mean}}}\,[ \% ]$	**2.8 (1.9)**	3.2 (2.0)	3.0 (1.8)	2.9 (1.7)	3.0 (1.7)
$\Delta {{\rm{DOS}}}\,[ \% ]$	50.8 (33.1)	55.7 (22.8)	19.6 (15.6)	20.3 (13.5)	**14.2 (14.5)**

The IQRs are given in parentheses, and the lowest absolute value for each metric is shown in bold.

### Evaluation of phonon-dependent thermodynamic and elastic properties

To assess how the improved phonon descriptions translate into physically relevant quantities, we extend the evaluation to the phonon-dependent properties, namely heat capacity at constant volume, vibrational part of the entropy and Helmholtz free energy. Furthermore, we calculate the elastic properties, such as the shear modulus and bulk modulus, and derive the Slack thermal conductivity^21^ from them. The temperature-dependent properties are calculated at 300 K.

**Fig. 3:**
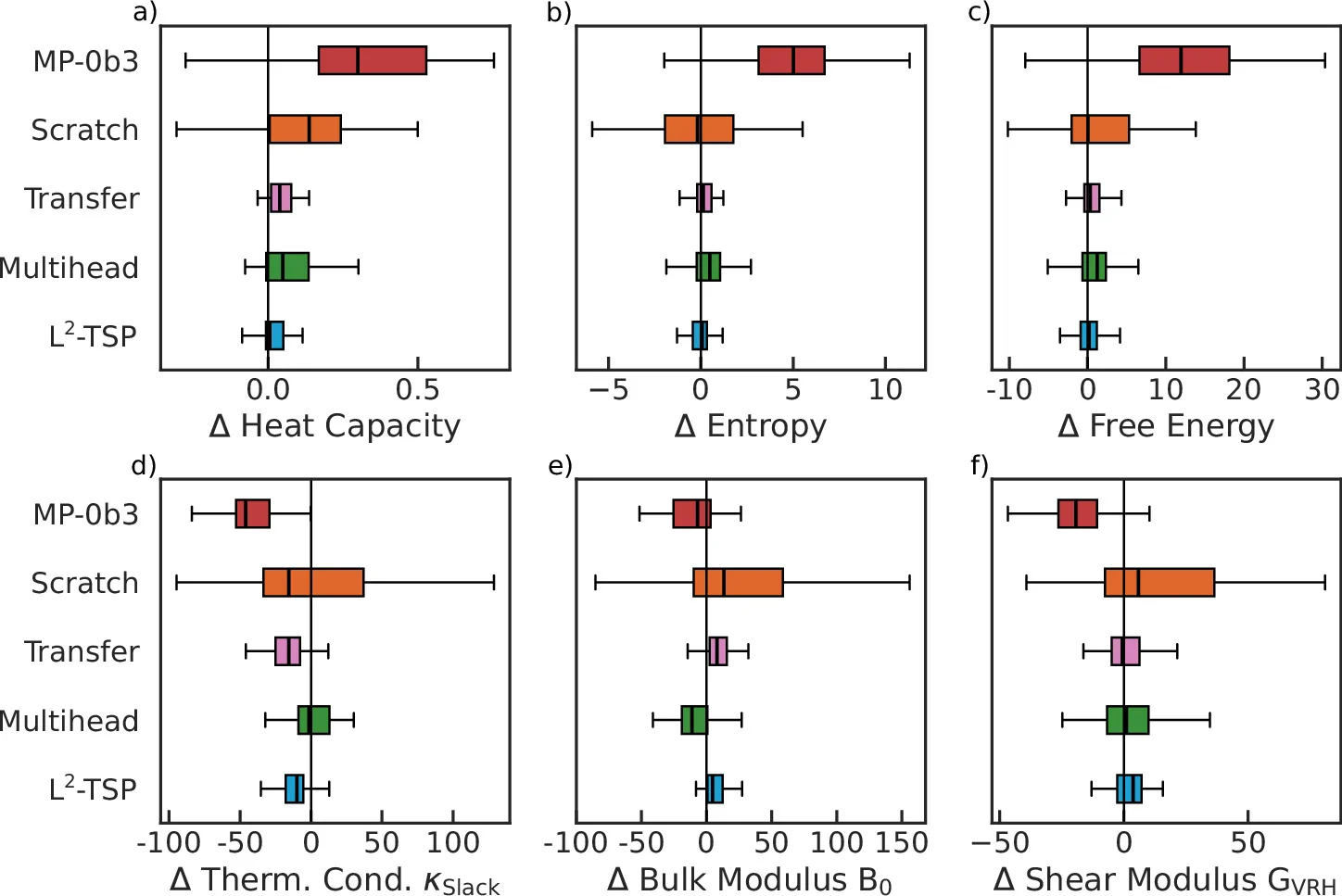
Deviations [%] of thermodynamic and elastic properties from the DFT reference results. **a** Heat capacity, **b** entropy, **c** vibrational free energy, **d** thermal conductivity, **e** bulk modulus, and **f** shear modulus are compared across the foundation model MP-0b3 (red), training from scratch (orange), transfer learning (violet), multi-head fine-tuning (green), and *L*^2^-TSP (blue). For better readability, outliers are not shown but are included in the SI Fig. S3.

We classify the Slack thermal conductivity as an elastic property here, since it depends on the average Grüneisen parameter, which we derive from the longitudinal and transversal sound velocities *v*_*L*_, *v*_*T*_^22^ obtained from elastic constants^22,23^ (see also SI Section “Calculation of physical properties”). In this sense, thermal conductivity, shear modulus, and bulk modulus provide a good test of how well the MACE models generalize, as they are sensitive to the quality of the energy predictions under small deformations such as compression or shear. Figure 3 summarizes the deviations of ML-predicted and DFT reference values. Across all phonon-dependent properties, fine-tuned models consistently outperform both the foundation MP-0b3 model and models trained from scratch. All fine-tuning strategies exhibit narrow error distributions with median deviations close to zero, indicating only small systematic bias. Among them, *L*^2^-TSP performs best overall, with the vast majority of materials falling within a  ± 5% error range across all phonon-dependent properties. Transfer learning achieves comparable accuracy, whereas the multi-head approach shows slightly larger dispersions. Since an additional replay set is used for multi-head fine-tuning to prevent catastrophic forgetting, we conclude that adding this data during fine-tuning is at least unnecessary and slightly counterproductive for predicting phonon-dependent properties. Interestingly, the from-scratch models show improvements over the foundation MP-0b3 model. This is in contrast to the earlier-found errors on the phonon band structure predictions themselves. It shows that band-structure errors do not directly translate into errors in thermodynamic properties.

Among the elastic properties, all fine-tuning models show improvements over the foundation MP-0b3 model. In contrast to the phonon-dependent properties, the poor performance of the from-scratch models indicates that elastic responses cannot be learned reliably from limited training data. Transfer learning and *L*^2^-TSP tend to underestimate thermal conductivity, corresponding to underestimated sound velocities and overestimated Grüneisen parameters. For the bulk and shear moduli, both methods yield comparable improvements, while the multi-head approach exhibits a slightly broader error distribution.

Across all thermodynamic and elastic properties, the foundation MP-0b3 model exhibits substantial systematic deviations. It systematically underestimates the thermal conductivity by around  − 50% on median, while it shows a wide error dispersion. From-scratch models show slightly improved performance on thermal properties but fail to predict elastic properties. They suffer from either systematic offsets or comparably large dispersions, making them much less reliable than any of the fine-tuning strategies. Overall, these results highlight that fine-tuning is the most reliable route to accurate thermodynamic and elastic properties, with *L*^2^-TSP and transfer learning providing the most robust and consistent performance.

### Imaginary mode prediction and phase transition

An even more stringent test of model fidelity is the prediction of dynamical instabilities. Phonons probe the curvature of the PES beyond the harmonic minimum and are directly linked to structural phase transitions. Identifying imaginary modes is particularly challenging because it requires a faithful description of shallow instabilities and anharmonic effects, and is therefore challenging not only for ML models but also for DFT calculations themselves. Computational inaccuracies can introduce non-physical instabilities^24^ and consequently, a direct comparison between DFT and ML predictions based solely on the existence of imaginary modes is delicate.

We first evaluate dynamical stability only at commensurate *q*-points, with a stringent cutoff frequency of  − 10^−4^ THz.

The DFT dataset contains nine unstable and 44 stable structures. The performance of the ML models in identifying unstable structures is shown in the confusion matrix in Table 3. Among all ML approaches, the *L*^2^-TSP strategy shows the best agreement with the DFT benchmark. For the unstable class, it achieves a precision of 100%, meaning no stable material is misclassified as unstable, and a recall of 89%, with only a single unstable material incorrectly predicted as stable. The multi-head fine-tuning models perform second best, incorrectly predicting two materials as stable and one as unstable. The foundation MP-0b3 model slightly outperforms the transfer learning models, while the from-scratch models show substantial difficulty in reproducing the correct stability classification. We conclude that additional training data, present either in the fine-tuned models due to the foundation model itself or during multi-head fine-tuning using replay data, has a beneficial effect on predicting instabilities. From-scratch models lack this additional data and therefore often fail to find alternative energy minima close to the relaxed structure. Transfer models tend to exhibit critical forgetting and, therefore, perform slightly worse than the other two fine-tuning approaches.

**Table 3:** Confusion matrix of stable or unstable structures in the dataset indicated by imaginary modes at commensurate *q*-points

	MP-0b3	Scratch	Transfer	Multihead	*L*^2^-TSP
DFT stable ML stable	42	43	43	42	44
DFT stable, ML unstable	2	1	1	2	0
DFT unstable, ML stable	2	6	3	1	1
DFT unstable, ML unstable	7	3	6	8	8
Precision (unstable)	78%	75%	86%	80%	100%
Recall (unstable)	78%	33%	67%	89%	89%

According to the DFT data, 44 structures are stable, while 9 are unstable. Precision and recall are given with respect to imaginary modes.

However, detecting an imaginary mode is not sufficient on its own. The most stringent question is whether the model also reproduces the correct anharmonic pathway and the final displacive phase. To address this, atoms are collectively displaced along selected imaginary modes, and the potential energy *U* is evaluated at different fixed mode amplitudes along the deformation, following the procedure proposed in ref.^25^. In this way, new energy minima and polymorphs can be identified. For the nine unstable materials, imaginary modes at high-symmetry points as well as band-structure minima are selected, and commensurate supercells are constructed only when their size remains below 1000 atoms. Because of the size restriction, DFT reference calculations are feasible for only 26 out of 41 (63%) considered *q*-points.

**Fig. 4:**
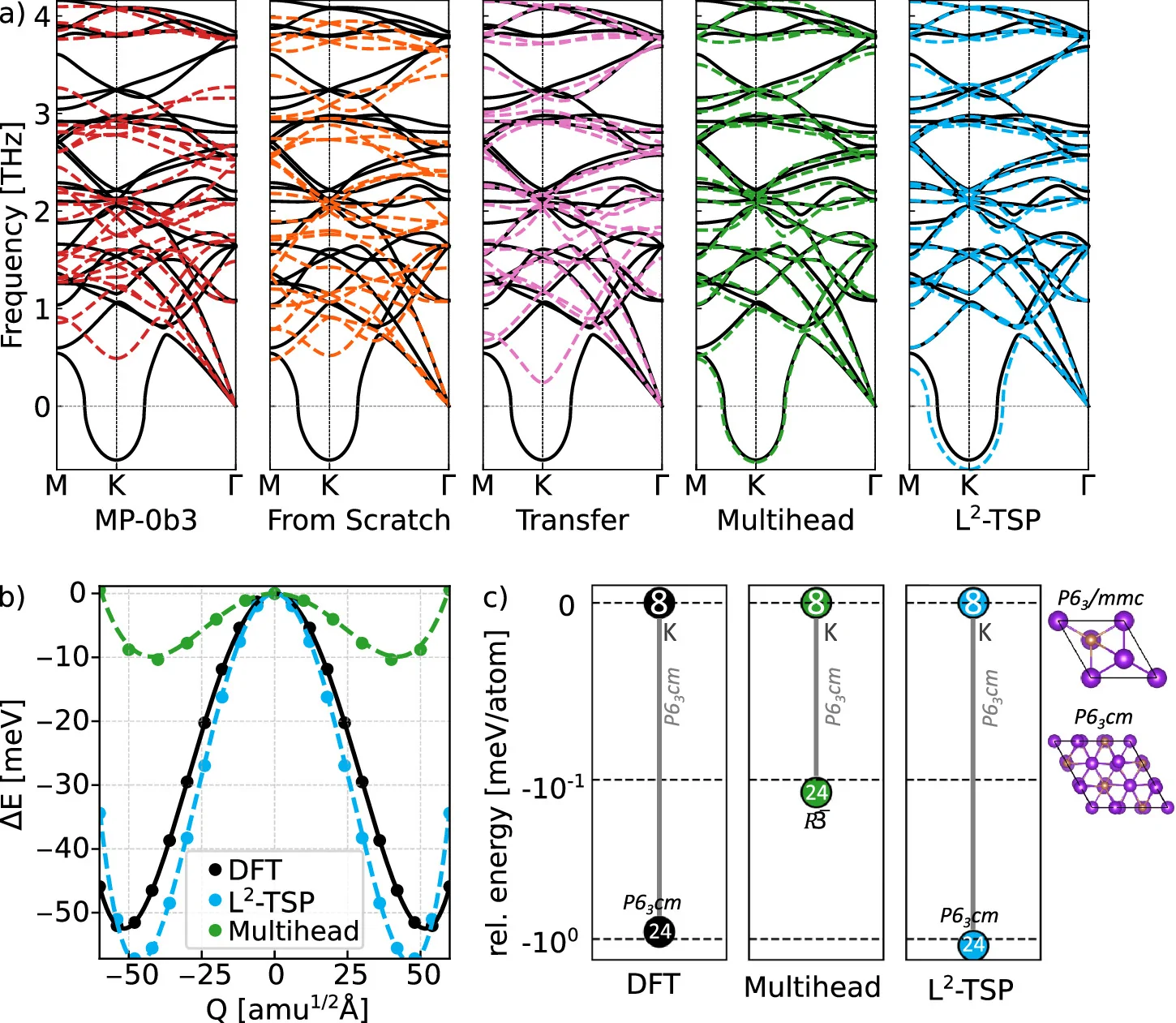
The K_3_Sb instability is traced from the phonon spectrum through the anharmonic potential to the resulting phase transition. **a** Section of the phonon band structure of K_3_Sb calculated with the ML models and compared with DFT (black). **b** Comparison of the anharmonic double-well potential along the *K* mode predicted by the multi-head fine-tuned model (green) and the *L*^2^-TSP model (blue). **c** Phase transition pathways (gray lines) predicted by the models that show imaginary modes. The numbers inside the circles indicate the number of atoms in the corresponding primitive unit cells. The initial and final phases are shown on the right.

K_3_Sb provides a representative example in which the relevant instability must be identified and followed to the correct polymorph. Figure 4(a) shows a section of the phonon band structure calculated with DFT and the ML models. The DFT reference exhibits a prominent imaginary mode at *K*. MP-0b3, from-scratch and transfer models fail to reproduce this instability, whereas the multi-head and *L*^2^-TSP models capture it correctly. By following the mode, we obtain the potential energy surface *U*(*Q*) along the normal coordinate *Q* as shown in Fig. 4(b). Both multi-head and *L*^2^-TSP reproduce the qualitative shape of the double-well potential, but differ noticeably in the depth and position of the minima. In both cases, the minima are shifted inward relative to DFT, while *L*^2^-TSP overestimates the energy difference and the multi-head model significantly underestimates it. The absence of an imaginary mode at *K* in the phonon band structures of the other models corresponds to a harmonic potential, which is inconsistent with the DFT reference. The crucial test, however, is whether ML models can correctly predict phase transition pathways. Relaxing the configurations in the energy minima of the double-well potentials leads to a new phase. For K_3_Sb, the phase transition pathway along *K* is shown in Fig. 4(c). Only the *L*^2^-TSP model recovers the relaxed structure in the same space group symmetry of *P*6_3_*cm*, while the multi-head model fails and relaxes to a different phase with space group symmetry $R\bar{3}$.

**Fig. 5:**
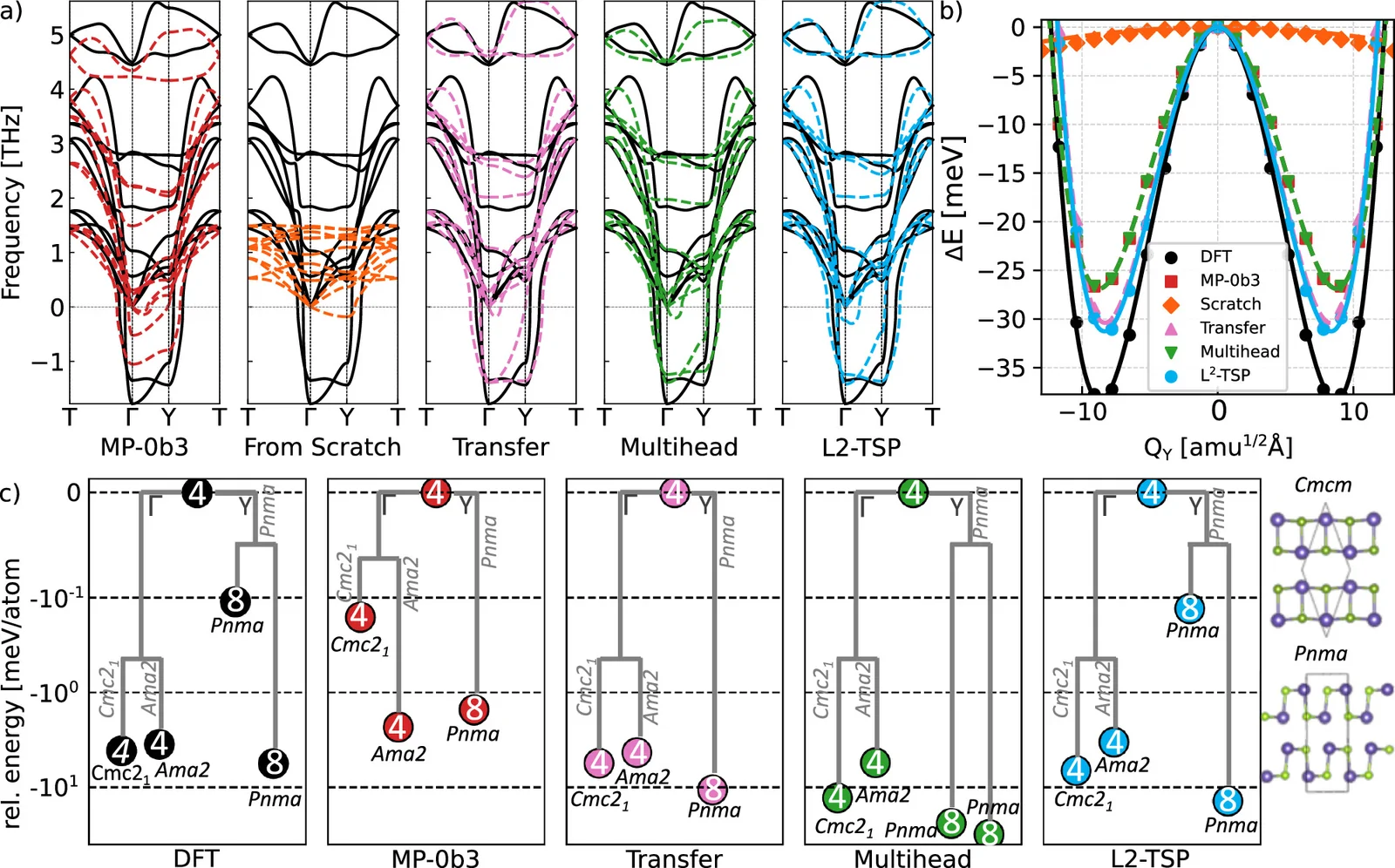
The Cmcm SnSe instability is traced from the phonon spectrum to its anharmonic potential and phase-transition pathways. **a** A section of the phonon band structure of *Cmcm* SnSe calculated with the different ML models compared to the DFT benchmark (black). **b** Comparison of the anharmonic double-well PES of the lowest *Y* mode predicted by MP-0b3 (red), the from-scratch model (orange), the transfer learning model (violet), the multi-head fine-tuned model (green) and the *L*^2^-TSP model (blue). **c** Phase transition pathways (gray lines) along the Γ and *Y* imaginary modes. The numbers inside the circles indicate the number of atoms in the corresponding primitive unit cells. The initial and final phases are shown on the right.

As a second example, Fig. 5 shows a section of the phonon band structure of SnSe together with the corresponding phase-transition pathways. The high-temperature *Cmcm* phase of SnSe is known to undergo a phonon-induced phase transition, leading to the low-temperature *Pnma* phase^26^. Here, all fine-tuned models are in good agreement with the DFT phonon benchmark. The from-scratch approach fails completely, and the foundation MP-0b3 model shows the characteristic softening in the higher phonon frequencies. All models show a small additional imaginary mode between Γ and *Y* which does not appear in the DFT calculation.

The potential energy *U* of the lowest *Y* mode is shown in Fig. 5(b). All ML models fail to capture the depth of the anharmonic potential, and the minima are shifted inwards. The from-scratch model does not reproduce the shape of *U* at all. For the DFT reference, we find that, at the Γ point, the unstable *Cmcm* phase relaxes into different symmetry groups *Cmc*2_1_ and *Ama*2 depending on the imaginary mode considered. Fig. 5(c) shows these phase transition pathways along the different modes at Γ and *Y*. The phase transition to the low-temperature phase *Pnma* is dominated by the smallest imaginary mode at *Y*^27^, which is reproduced by all models. *L*^2^-TSP shows the best agreement in energy of the individual phases.

Finally, we assess the models more generally by quantifying how many displacive phase transitions can be reproduced across the nine preselected unstable materials. For each material, the set of final relaxed phases predicted with the ML models is compared to the corresponding DFT benchmark set. Prior to the evaluation, all phases equivalent to the initial phase are removed, because they represent no new transition. Cases in which DFT exhibits imaginary modes due to interpolation errors within a harmonic potential, while the ML model predicts no instability and therefore provides the physically correct description of the potential energy surface, are excluded from the analysis. A phase predicted by the ML model is counted as a true positive if it matches a DFT benchmark phase in both space group and number of atoms in the primitive unit cell. Additionally, phases predicted by the ML model but absent in the DFT benchmark are classified as false positives, whereas DFT phases not recovered by the ML model are counted as false negatives. Further details are provided in SI Section “Phase transition pathways”, including a specific example of interpolation errors shown in SI Fig. S4. Detailed phase transition pathways obtained from DFT and each ML approach are provided in SI Tables S2–S7.

The resulting precision, recall, and F1 score for the fine-tuning strategies over the set of the nine non-stable structures are presented in Table 4. Among the fine-tuned approaches, *L*^2^-TSP yields the best overall performance, with a precision of 68%, a recall of 65%, and an F1 score of 0.66. Its performance is therefore slightly better than that of the foundation model MP-0b3, which achieves a higher recall of 66% but a lower precision of 60%, indicating that it reproduces slightly more benchmark phases at the expense of more false-positive predictions. In contrast, the other fine-tuning strategies can not compete with these results. These results suggest that *L*^2^-TSP best preserves the foundation model’s ability to generalize to unseen phases while still benefiting from system-specific fine-tuning. Conventional transfer learning appears to reduce generalization capability, likely due to catastrophic forgetting. Surprisingly, the multi-head approach performs even worse, although the additional replay dataset during fine-tuning is intended to mitigate forgetting of the PES beyond the fine-tuning structures. A possible explanation is that the replay set of 100 structures is too small. However, increasing the size would also increase the computational costs of fine-tuning.

**Table 4:** Recall, precision, and F1 scores of new phase predictions for different fine-tuning approaches

	MP-0b3	Scratch	Transfer	Multihead	*L*^2^-TSP
Precision	60%	33%	54%	38%	**68%**
Recall	**66%**	33%	58%	50%	65%
F1	0.63	0.33	0.56	0.43	**0.66**

For each score, the highest value across all fine-tuning approaches is highlighted in bold.

Precision and recall are calculated separately for each material and then averaged based on the number of materials evaluated.

### Computational cost

**Fig. 6:**
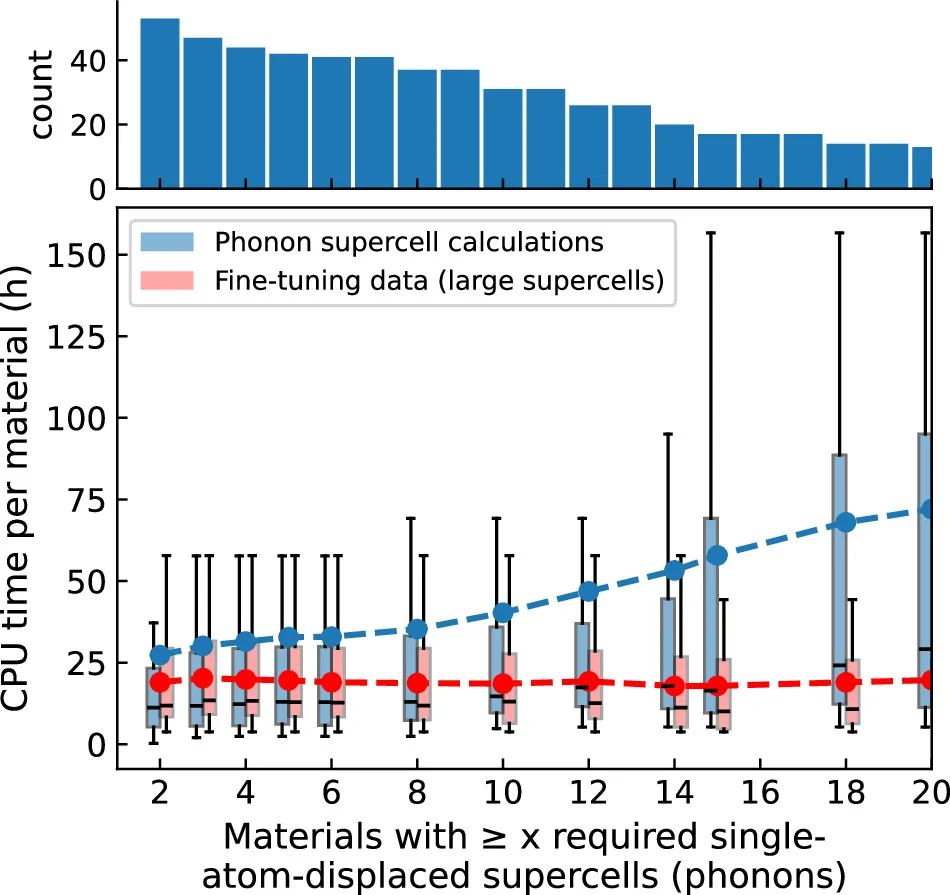
The computational advantage of fine-tuning increases with the number of displaced supercells required for DFT phonon calculations. Average computational cost per material as a function of the minimum required number of single-atom displaced supercells for phonon calculations. The x-axis shows cumulative subsets of materials that require at least a given number of displaced supercells. The blue boxplots correspond to DFT phonon supercell calculations, while the red boxplots indicate the computational cost of generating the large supercell fine-tuning data. The blue and red dashed lines show the mean values. The histogram shows the number of materials counted in each batch.

The computational cost is dominated by the generation of DFT single-atom-displaced supercells required for the phonon calculation, averaging 27.3 h per material (computed using three nodes with 48 CPU cores each). In comparison, generating 10 large supercell training structures for fine-tuning requires 18.8 h per material on average, resulting in an overall reduction in computational time of 32%. This difference reflects the larger size of the single-atom displaced phonon supercells, which have edge lengths above 20 Å, whereas the large training supercells are typically limited to 15 Å. The computational cost of phonon calculations depends strongly on the symmetry of the system, which determines the number of required single-atom-displaced supercells. Figure 6 shows that the benefit of fine-tuning increases for materials requiring at least a given number of single-atom-displaced supercells. For materials requiring 10 or more single-atom-displaced supercells, the total computational time is reduced by 54% on average, increasing to 92% for systems requiring 20 or more displaced supercells. The blue boxplots further show that the spread in CPU time grows with increasing structural complexity. The median runtime for generating single-atom-displaced supercells and fine-tuning data begins to diverge noticeably for materials requiring 12 or more single-atom-displaced supercells. The strongly elevated mean values indicate the presence of outliers that require substantially longer phonon calculations. It is precisely for these cases that the fine-tuning approaches become particularly advantageous.

All model training procedures are significantly cheaper, ranging from 1.2 min in scratch and transfer fine-tuning to 7.3 min per material for *L*^2^-TSP fine-tuning and 8.3 min per material for multi-head fine-tuning with a set of 100 replay structures on a NVIDIA A100 GPU.

### Summary

We systematically evaluated different fine-tuning strategies based on the MACE-MP-0b3 foundation model across phonons, thermal and elastic properties, and phase-transition behavior. Our results show that as few as 10 additional fine-tuning structures are sufficient to substantially improve the predictive performance of the MLIP for a given material. All investigated fine-tuning strategies, i.e., transfer learning, multi-head fine-tuning, and the *L*^2^-TSP approach, consistently enhance the accuracy of phonon band structures and derived thermal properties compared to both the foundation model and from-scratch training. For elastic properties, a similar trend is observed. With respect to imaginary phonon modes, *L*^2^-TSP and multi-head fine-tuning improve instability prediction across the dataset, whereas conventional transfer learning shows reduced performance, which may be related to catastrophic forgetting. However, a layer-selected transfer-learning configuration with comparable harmonic phonon accuracy reproduces the dynamical-stability classification of *L*^2^-TSP, demonstrating that the choice of trainable layers can strongly affect instability prediction. A more detailed analysis reveals that imaginary-mode predictions alone are not a sufficient indicator of accurate PES prediction. By explicitly evaluating displacive phase transitions, we find that most fine-tuning configurations struggle to generalize across the PES and perform worse than the foundation model. Notably, the layer-selected transfer-learning configuration also remains below the foundation model in phase-recovery performance despite matching *L*^2^-TSP in dynamical-stability classification. In contrast, *L*^2^-TSP achieves the highest overall phase-recovery performance among the investigated approaches, indicating that improvements in harmonic phonon accuracy and dynamical-stability prediction do not necessarily translate into a faithful description of the broader PES.

## Methods

### Reference calculations (DFT)

All DFT calculations were performed using VASP^28–30^ with the PBE exchange-correlation functional^31^ and with the PAW framework^32,33^. Unless stated otherwise, an energy convergence criterion of 10^−8^ eV, a plane-wave cutoff of 520 eV, and a *k*-point spacing of 0.1 Å^−1^ were used. Structural relaxations employed a force threshold of 0.5 meV/Å.

Harmonic phonons were computed using Phonopy^12,13^ with the PhononMaker workflow in atomate2^23^. Cubic supercells with a minimum side length of 20 Å were used, sampled at the Γ point only. Force constants were extracted using symfc^34^. Elastic constants were calculated using the ElasticMaker workflow in atomate2^23^ with a force threshold of 1 meV/Åand a fixed symmetry during the initial structure relaxation.

Equilibrium volumes were scaled between  ± 5% in steps of 1%, followed by atomic relaxation at fixed volume. Energy-volume data were fitted to the Birch-Murnaghan equation of state^35^.

### Generation of fine-tuning data

Datasets of primitive unit cells, small and large supercells for fine-tuning were generated using a systematic rattling protocol. Small supercells were constructed as cubic cells with sizes ranging between (5 Å)^3^ and (10 Å)^3^, while the large cubic supercells ranged from (10 Å)^3^ to (15 Å)^3^. For each case, five rattled configurations with a mode amplitude of  ~ 0.1 Åwere created by using the Monte Carlo rattling procedure implemented in the Hiphive code^14^. The lattice of each rattled structure was further perturbed by randomly scaling the angles and vectors within  ± 2% range. Finally, the volumes were uniformly scaled by  − 5%,  − 2.5%, 0%,  + 2.5%, and  + 5% and were then relaxed with the FIRE optimizer^36^ using the MACE-MP-0b3 foundation model^1^ until the forces converged to 10^−4^ eV/Å. From each relaxation trajectory, four configurations equally spaced in energy from the starting structure until 90% of convergence were selected for subsequent DFT calculations. In total, 20 configurations were obtained per material. The 4 configurations originating from the (0%) unscaled volume trajectory were assigned as validation data, while the remaining 16 configurations were used for training. The fine-tuning data were successively added along the four relaxation trajectories, starting with only the outermost structures from each trajectory. In subsequent steps, the next inner configurations were also included, progressively expanding the training set toward the equilibrium region. Consequently, the first training data points correspond to the  ± 5 % volume-scaled and distorted structures, followed by increasingly relaxed configurations that approach the energy minimum.

### Model hyperparameters

In this study, we use the MACE-MP-0b3 foundation model, which employs a cutoff radius of 6 Å and two interaction layers, limiting the receptive field to 12 Å. Models were fine-tuned using the AdamW optimizer with a ReduceLROnPlateau scheduler for up to 200 epochs. For all training approaches, the model checkpoint with the lowest validation loss was retained for subsequent evaluation. Training minimized a weighted sum of Huber losses for energies, forces, and stresses with (*w*_*e*_, *w*_*f*_, *w*_*s*_) = (10, 100, 1000) and *δ*_*H*_ = 0.01. Transfer-learned models used (*lr*, *wd*) = (0.01, 5 × 10^−3^), multi-head models used (0.01, 5 × 10^−7^) together with a replay dataset of 100 randomly chosen structures, and Equitrain models (0.01, 10). The from-scratch models are not pre-trained; instead, they are trained from scratch on the fine-tuning data using the same training parameters as the multi-head models.

### Fine-tuning strategies

Equitrain^15^ implements multiple fine-tuning strategies, including layer freezing, *L*^2^ starting point (*L*^2^-SP)^10^, and Low-Rank Adaptation (LoRA)^11^. In the present study, we combine *L*^2^-SP with selective layer fine-tuning, a strategy we refer to as targeted *L*^2^-SP (*L*^2^-TSP).

In standard fine-tuning, the parameters of a pre-trained model are initialized from the pre-trained weights and subsequently optimized on the target dataset. When only a small amount of fine-tuning data is available, unconstrained optimization may lead to substantial deviations from the pre-trained solution and consequently to a loss of previously learned information. *L*^2^-SP addresses this by explicitly regularizing the model parameters toward their pre-trained values^10^. For a pre-trained weight matrix *W*_0_, the fine-tuned weights *W* are obtained by minimizing

1 $$W=\arg {\min}_{W} \, ({{\mathcal{L}}} (W)+ \lambda {\parallel} W-{W}_{0} {\parallel}_{F}^{2}),$$

where ${{\mathcal{L}}}$ denotes the fine-tuning objective and *λ* controls the strength of the regularization. In contrast to conventional *L*^2^ weight decay, which penalizes the magnitude of the weights themselves, *L*^2^-SP penalizes their displacement from the pre-trained solution.

In Equitrain, this objective is implemented using an additive residual parameterization,

2 $$W={W}_{0}+\Delta W,$$

where *W*_0_ remains fixed and only the residual weights *ΔW* are optimized. Applying weight decay exclusively to *ΔW* gives

3 $${\Delta} {W} =\arg {\min }_{{\Delta} W} \, \left({{\mathcal{L}}}({W}_{0}+ {\Delta} {W} )+\lambda {\parallel} {\Delta} W {\parallel} _{F}^{2}\right).$$

Since *ΔW* = *W* − *W*_0_, this formulation is equivalent to the *L*^2^-SP objective above. The residual parameterization therefore provides a direct implementation of regularization toward the pre-trained starting point while keeping the reference parameters *W*_0_ unchanged. In practice, we use* AdamW*, which applies weight decay to the residual parameters ΔW separately from the adaptive updates computed from the fine-tuning loss.

In *L*^2^-TSP, we additionally restrict the additive updates to a selected subset of the network, while all remaining parameters are kept fixed. Selective, or “surgical”, fine-tuning has previously been shown to improve adaptation under distribution shifts, with the optimal subset of trainable layers depending on the type of shift^17^. For the MACE-MP-0b3 model considered in the main text, we apply *L*^2^-SP only to the first two layers of the network, corresponding to the node embedding and the first interaction layer. This choice yields the lowest phonon error among the layer subsets investigated in this work (see Supplementary Information). Thus, *L*^2^-TSP combines regularization toward the pre-trained solution with targeted adaptation of the early representation-building layers.

LoRA^11^ is related in that it also keeps *W*_0_ fixed and represents adaptation through an additive update *ΔW*. However, LoRA further constrains the update by factorizing it as

4 ΔW=BA,

with $A\in {{\mathbb{R}}}^{r\times {d}_{{{{{\rm{in}}}}}}}$ and $B\in {{\mathbb{R}}}^{{d}_{{{{{\rm{out}}}}}}\times r}$. Typically, $r\ll \min ({d}_{{{{{\rm{in}}}}}},{d}_{{{{{\rm{out}}}}}})$, such that the number of trainable parameters is substantially reduced. The principal distinction is therefore that *L*^2^-SP constrains adaptation through an explicit penalty on the distance from the pre-trained solution, while *L*^2^-TSP additionally restricts which parameters can be adapted. LoRA instead constrains the structure and dimensionality of the additive update through its low-rank parameterization.

For the main analysis in this study, we employ *L*^2^-TSP as described above and as implemented in Equitrain. To assess the effect of low-rank adaptation separately, we additionally compare against LoRA models with different ranks using the LoRA implementation in MACE.

### Thermodynamic and elastic property evaluation

Phonon dispersions and thermodynamic properties at 300 K were computed using Phonopy^12,13^. A 20 × 20 × 20*q*-point mesh was used for the Brillouin-zone integration of the thermodynamic properties.

Elastic constants and equations of state were computed using the corresponding ML models following the same protocols as for the DFT reference. In rare cases, ML-based equation-of-state fits found individual E(V) points to lie far from the physically expected energy-volume curve. These outliers were excluded from the Birch-Murnaghan equation-of-state fit.

### Structural instabilities and phase transitions

Dynamical instabilities were identified by imaginary phonon frequencies below  − 10^−4^ THz at commensurate *q*-points. Supercells commensurate with unstable wave vectors were displaced along the corresponding eigenmodes, and a set of static calculations was performed to find the minima. In case of degenerate eigenvalues, a linear combination of eigenvectors was used. The maximum eigenvalue multiplicity in the dataset was four. For one-dimensional mode subspaces, 21 static energy calculations were performed, while two-, three-, and four-dimensional subspaces were sampled on uniform grids of 21 × 21, 9 × 9 × 9, and 7 × 7 × 7 × 7 points, respectively. Local energy minima within each grid were identified, and the corresponding configurations were subsequently relaxed by geometric optimization. The DFT calculations were carried out with an energy convergence criterion of 10^−7^ eV and a cutoff energy of 400 eV. Structural optimization with the ML models was performed until the ionic forces converged below 5 meV/Å. The geometric optimization of the ML models was additionally limited to a maximum of 2000 steps. During relaxation, symmetry lowering was forbidden. Final symmetries were determined using spglib^37^ with a tolerance of 0.1 Å.

## Supplementary information

Supplementary Information

## Data Availability

The datasets, evaluation and fine-tuning scripts, and machine-learning models used in this work are available in a Zenodo repository at https://doi.org/10.5281/zenodo.22180297. The Equitrain software package used to implement L2-SP and L2-TSP fine-tuning is available at https://github.com/BAMeScience/equitrain. Version 0.1.0 was used for the calculations presented in this study. Detailed instructions for reproducing the Equitrain L2-TSP fine-tuning workflow used in this study with the current Equitrain version are available at https://bamescience.github.io/equitrain/phonon-finetuning-paper.
