# Supplementary material for: Targeted fine-tuning of machine-learning interatomic potentials for phonons and phase transitions
Source: NPJ Comput Mater. 2026 Oct 7;12(1):354. doi: 10.1038/s41524-026-02338-w (PMC13713550; doi:10.1038/s41524-026-02338-w)
Supplement: Supplementary file 1 [file 41524_2026_2338_MOESM1_ESM.pdf]

SUPPLEMENTARY INFORMATION

Targeted Fine-Tuning of Machine-Learning Interatomic Potentials for Phonons and Phase Transitions

Jonas Grandel, Philipp Benner, Janine George

Contents

Foundation model comparison 1
Calculation of physical properties 5
Phase transition pathways 8
Comparison of L^2-TSP with LoRA 15
Effect of layer selection in transfer learning and L^2-SP fine-tuning 15

Foundation model comparison

At the time this study was conducted, MP-0b3 was the most recent foundation model available. However, within the MACE-family of foundation models, MACE-MP-0b3, MACE-MPA-0 [1], and MACE-OMAT-0 [2] are the currently available state-of-the-art models. The MACE-F model is a model specifically trained for phonon calculations [3]. To assess the impact of foundation model choice, we evaluate the phonon band structures of the materials in our dataset using these foundation models. As summarized in Table S1, MACE-MPA-0

Table S1: Phonon MAE and IQR in THz of four different foundation models.

MP-0b3	MPA-0	MACE-OMAT-0	MACE-F
0.27 (0.18)	0.16 (0.19)	0.10 (0.09)	0.29 (0.23)

and MACE-OMAT-0 exhibit much lower error than MP-0b3, confirming that the latter is no longer the strongest available pre-trained model. A key reason for the improved performance of the newer models is the increase in training set sizes. Whereas MP-0b3 was trained on the Materials Project dataset with a total of 1.58M structures, MACE-MPA-0 was trained on an additional 10.4M structures. MACE-OMAT-0 was trained on the OMat24 dataset containing 101M structures. However, the L^2-TSP fine-tuning strategy reduces the phonon error to a median MAE (IQR) of 0.05 (0.05) THz, substantially outperforming all tested models. This demonstrates the immense potential of fine-tuning, which can also be applied to the other models in precisely the same way, probably leading to even smaller errors. The gains from material-specific fine-tuning exceed the differences among the available foundation models. The present results likely represent a conservative estimate of the performance

that can be achieved with fine-tuning and especially with L^2-TSP. Notably, MACE-F shows a larger error than the other models, despite being designed for phonon calculations. This further highlights the superiority of material-specific fine-tuning.

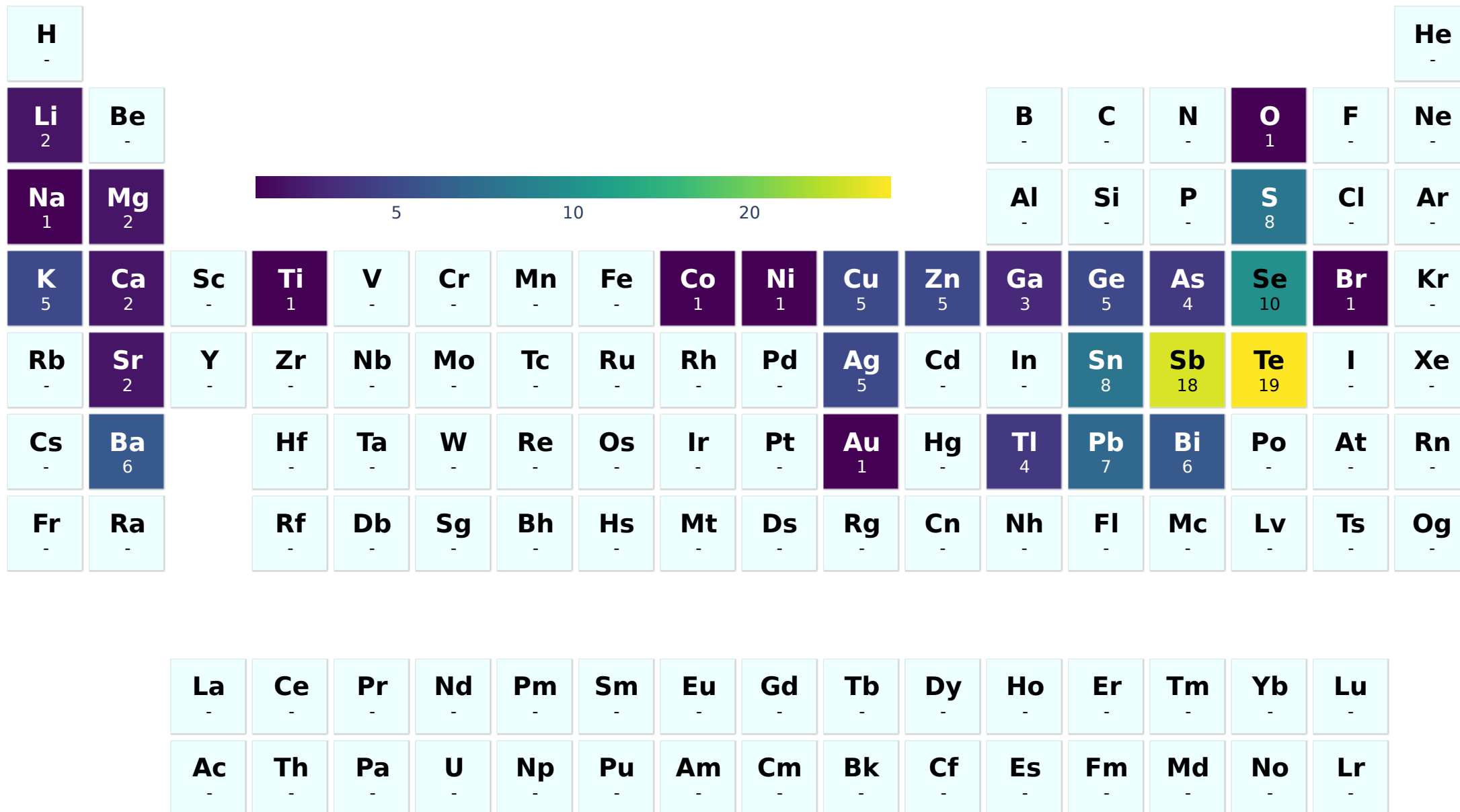

Figure S1: **Heatmap of elements contained in the material dataset**. The heatmap was created with [4].

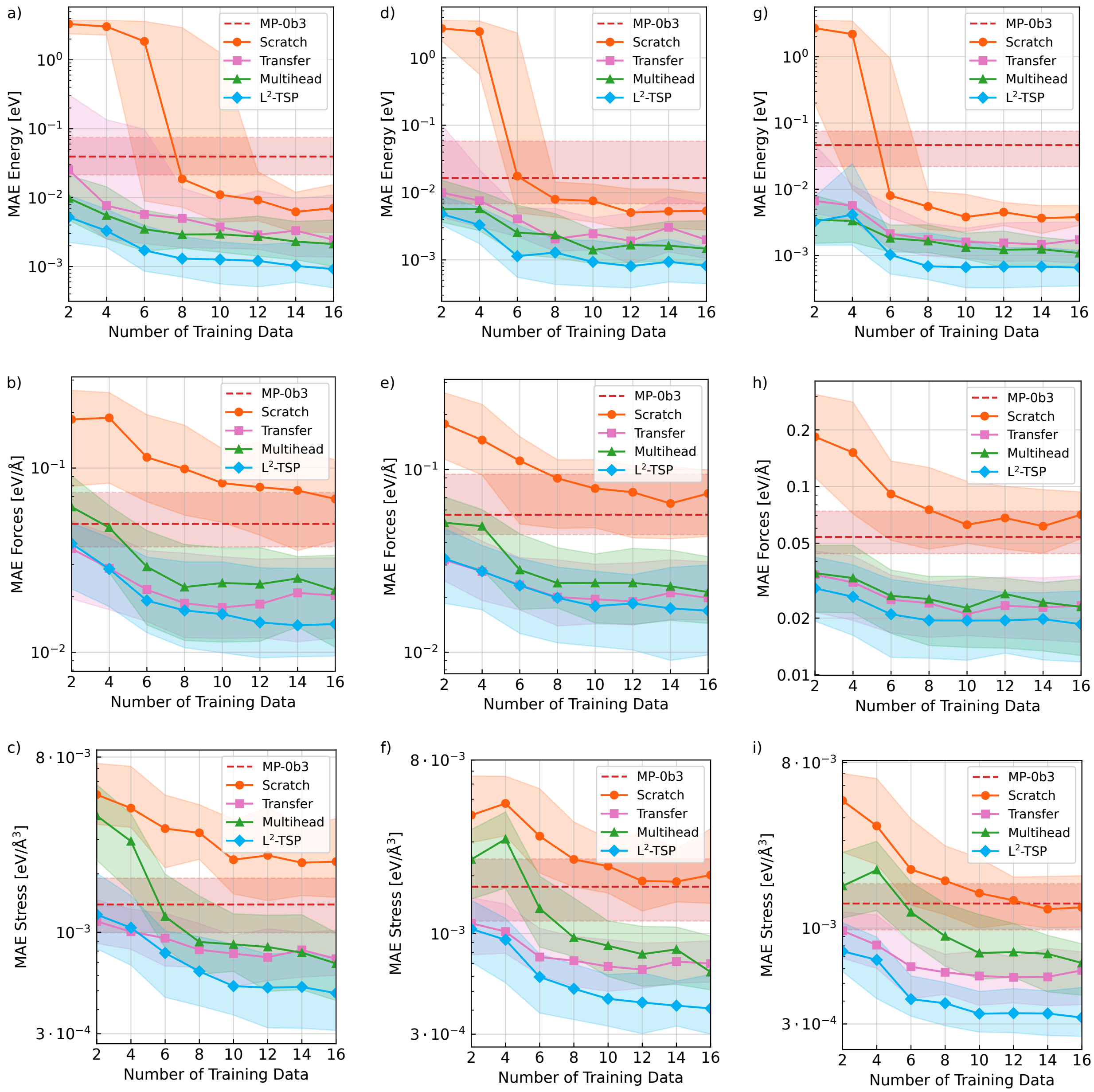

Figure S2: **Energy, force, and stress errors converge with increasing fine-tuning data across all training structure sizes.** Median MAE and inter-quartile range on the validation sets of all 53 materials for energy, force and stress with models trained using with a)–c) primitive unit cells, d)–f) small supercells, and g)–i) large supercells. Models trained from scratch (orange circles), transfer learning (violet squares), multi-head fine-tuning (green triangles), and L^2-TSP (blue diamonds) are shown. For comparison, the red dashed line shows how well the foundation MP-0b3 model performs on the validation sets. The overall convergence trend is the same in all figures. The MAE decreases significantly with up to 10 additional fine-tuning data points. After that, the trend flattens out.

Calculation of physical properties

The heat capacity, vibrational Helmholtz free energy, and entropy are calculated directly from the phonon band structures using the implementation provided in the Phonopy code [5]. For all calculations, imaginary frequencies were excluded.

Heat capacity

The heat capacity at constant volume C_V is defined as

$$C_V = \left(\frac{\partial U}{\partial T}\right)_V \tag{S1}$$

where the internal vibrational energy is given by

$$U = \sum_{k,j} \hbar\omega_j(\mathbf{k}) \left(\frac{1}{2} + g(\hbar\omega_j(\mathbf{k})\right) \tag{S2}$$

and g being the Bose-Einstein distribution.

Vibrational Helmholtz free energy

The Helmholtz free energy F is calculated solely from the phonon frequencies according to

$$F = -k_B T \ln(Z) \tag{S3}$$

by using the partition function

$$Z = \prod_{k,j} \frac{\exp(-\frac{1}{2}\beta\hbar\omega_j(\mathbf{k}))}{1 - \exp(-\beta\hbar\omega_j(\mathbf{k}))} \tag{S4}$$

and $\beta = 1/k_B T$.

Entropy

The entropy S is defined as

$$S = -\frac{\partial F}{\partial T} \tag{S5}$$

Slack thermal conductivity

The Slack thermal conductivity [6]

$$\kappa_{\text{Slack}} = A(\gamma)\frac{M\delta n^{1/3}\Theta_D^3}{\gamma^2 T} \tag{S6}$$

depends on the average Grüneisen parameter γ, Here, γ is calculated from the longitudinal and transversal sound velocities v_L, v_T [7]

$$\gamma = \frac{3}{2}(\frac{1+\nu}{2-3\nu}), \qquad \nu = \frac{1-2(v_T/v_L)^2}{2-2(v_T/v_L)^2} \tag{S7}$$

The sound velocities are obtained from elastic-property calculations [7, 8]. They are calculated from the Voigt-Reuss-Hill average shear modulus $\bar{G}_{\mathrm{VRH}}$ and bulk modulus $\bar{B}_{\mathrm{VRH}}$

$$v_T = \left(\frac{\bar{G}_{\mathrm{VRH}}}{\rho_m}\right)^{\frac{1}{2}} \tag{S8}$$

$$v_L = \left(\frac{\bar{B}_{\mathrm{VRH}} + 4/3\bar{G}_{\mathrm{VRH}}}{\rho_m}\right)^{\frac{1}{2}} \tag{S9}$$

where ρ_m is the mass density.

Bulk modulus

The bulk modulus was calculated by fitting the Birch-Murnaghan equation of state [9] to energy-volume data.

$$E(\eta) = E_0 + \frac{9B_0V_0}{16}(\eta^2 - 1)^2(6 + B_0'(\eta^2 - 1) - 4\eta^2) \tag{S10}$$

with $\eta = (V/V_0)^{\frac{1}{3}}$. Here, V, V_0 are the volume and equilibrium volume, E_0 is the minimum energy, B_0 is the bulk modulus, and B_0' is its first derivative with respect to pressure.

Shear modulus

The Voigt-Reuss-Hill shear modulus G_{VRH} is defined as the average of the Voigt shear modulus G_V and the Reuss shear modulus G_R.

$$G_{\mathrm{VRH}} = \frac{1}{2}(G_V + G_R) \tag{S11}$$

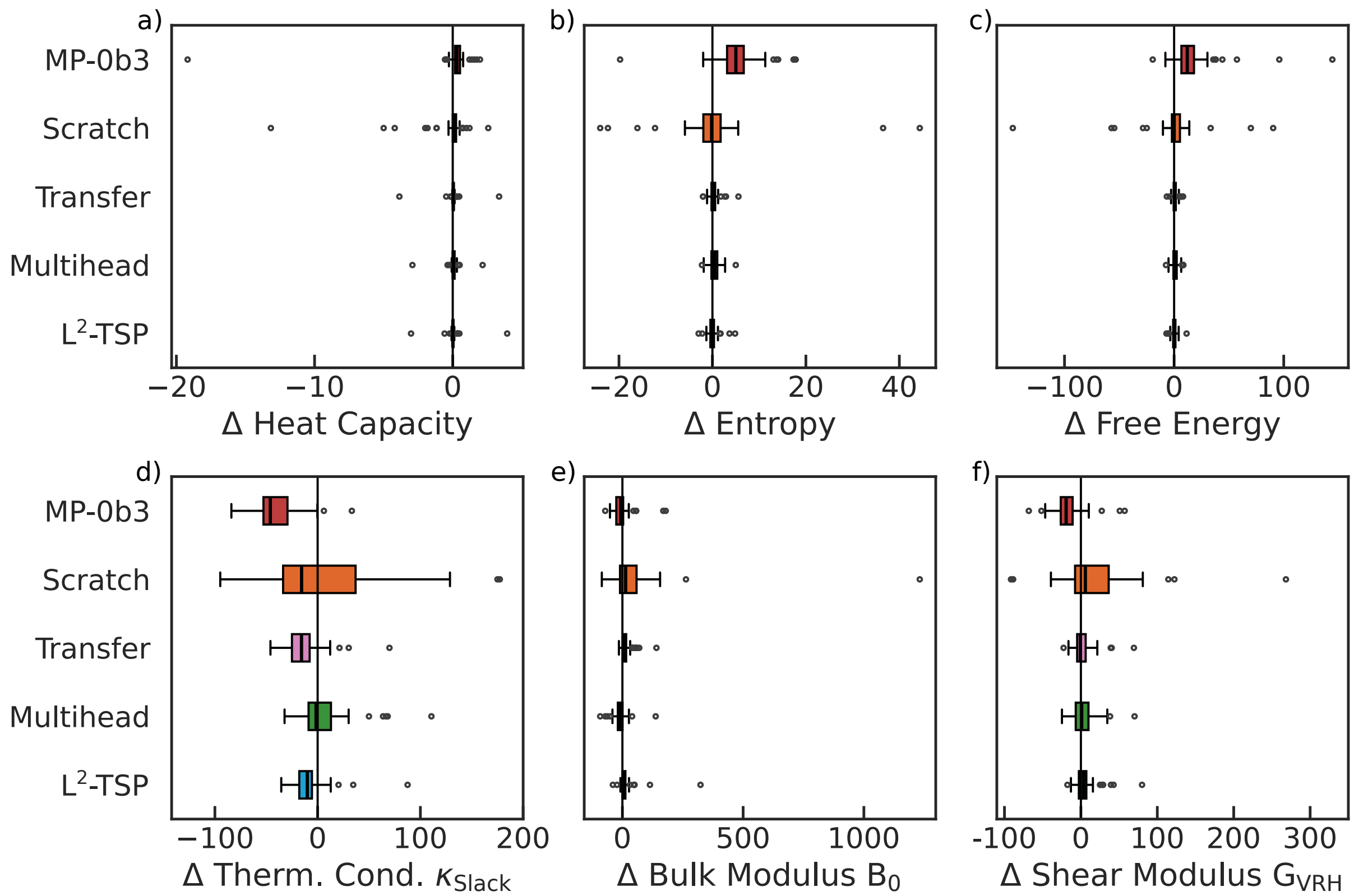

Figure S3: **The full error distributions of the predicted thermodynamic and elastic properties, including outliers.** Relative Deviations [%] of (a)heat capacity, (b) entropy, (c) vibrational free energy, (d)thermal conductivity, (e)bulk modulus, and (f) shear modulus are compared across the foundation model MP-0b3 (red), training from scratch (orange), transfer learning (violet), multi-head fine-tuning (green), and L^2-TSP (blue). Boxplots including outliers are shown. In most cases, the outliers appear mainly in the MP-0b3 and from-scratch, whereas all fine-tuning approaches reduce the number of outliers.

Phase transition pathways

Within the finite-displacement approach, phonon band structures are obtained exactly only at q-points that are commensurate with the chosen supercell. The size and shape of the supercell used for single-atom displacements, therefore, directly determine which q-points are calculated exactly and which frequencies are obtained by Fourier interpolation. To analyze the phase transition pathways, all imaginary modes at high-symmetry points and at minima of the phonon bands were considered. For each of these modes, the smallest supercell commensurate with the corresponding q-point was constructed. Only calculations with fewer than 1000 atoms per supercell were carried out. By calculating the potential energy along the corresponding imaginary modes and comparing this to ML predictions, we identify four possible scenarios.

1. DFT and ML predict identical phase transition pathways. The shapes of the energy potential and the relaxed structure obtained at the energy minimum are consistent between the two methods.

2. The DFT phonon band structure exhibits imaginary modes, but the energy potential along the corresponding mode remains purely harmonic. This behavior can be attributed to interpolation errors inherent to the finite-displacement approach.

   An example is shown in Figure S4 (a). The q-point $R = (0, 0.5, 0.5)$ is not commensurate with the chosen supercell matrix

   $$S_1 = \begin{pmatrix} 5 & -2 & 0 \\ 5 & 2 & 0 \\ 0 & 0 & 5 \end{pmatrix} \tag{S12}$$

   The corresponding energy potential along the degenerate modes is shown in Figure S4 (b). The black dots represent the calculated DFT energies, while the colored surface shows the fitted harmonic energy function

   $$E(Q_0, Q_1) = -65.59199 \text{ eV} + 9.8 \cdot 10^{-5} \frac{\text{eV}}{\text{amuÅ}^2} Q_0^2 + 9.8 \cdot 10^{-5} \frac{\text{eV}}{\text{amuÅ}^2} Q_1^2 \tag{S13}$$

   with $R^2 = 0.99$. By choosing a different supercell matrix

   $$S_2 = \begin{pmatrix} 5 & -2 & 0 \\ 5 & 2 & 0 \\ 0 & 0 & 4 \end{pmatrix} \tag{S14}$$

   the single-atom displaced supercells become commensurate with R. The resulting phonon band structure, shown in Figure S4 (c), confirms that the imaginary mode in (a) originated from Fourier interpolation errors.
   The q-point (0.02,0.02,0) between S and T would require a large supercell of size $50 \times 50 \times 1$ to calculate the potential energy, which is computationally prohibitive and therefore not tested.

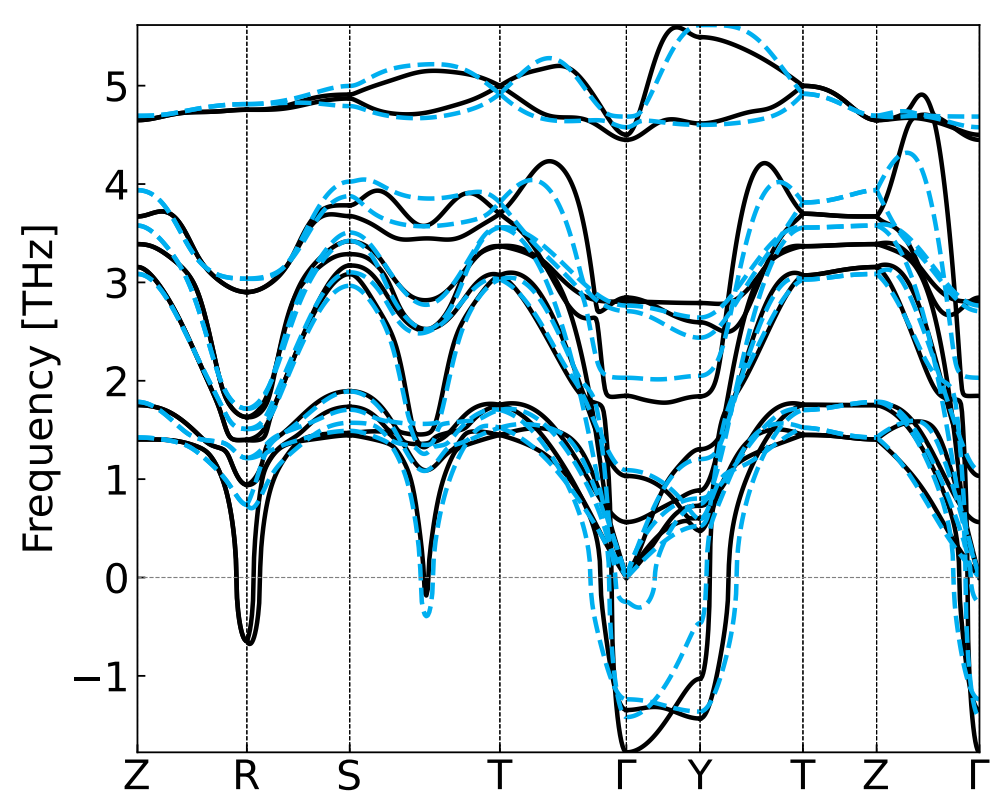

(a)

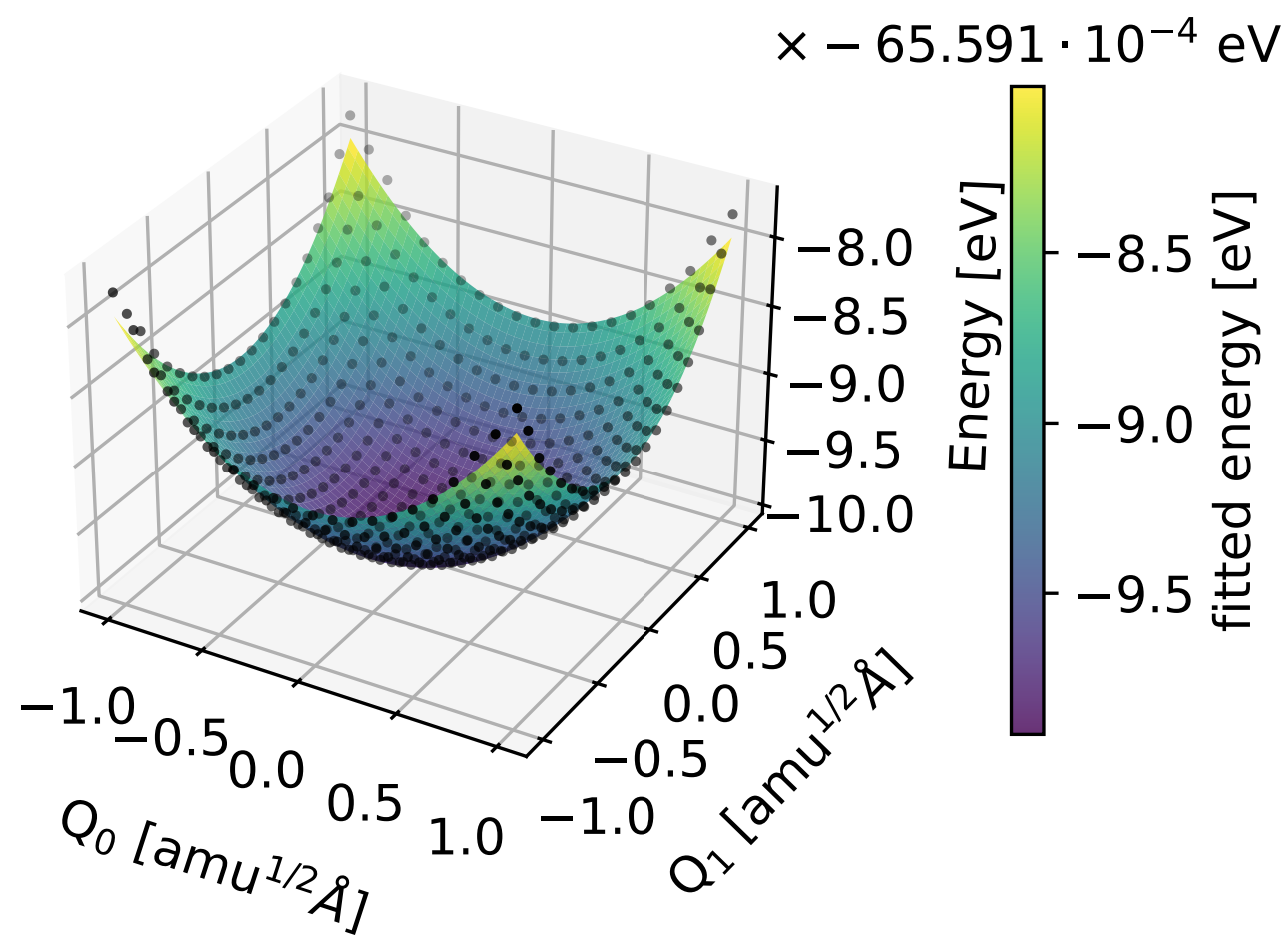

(b)

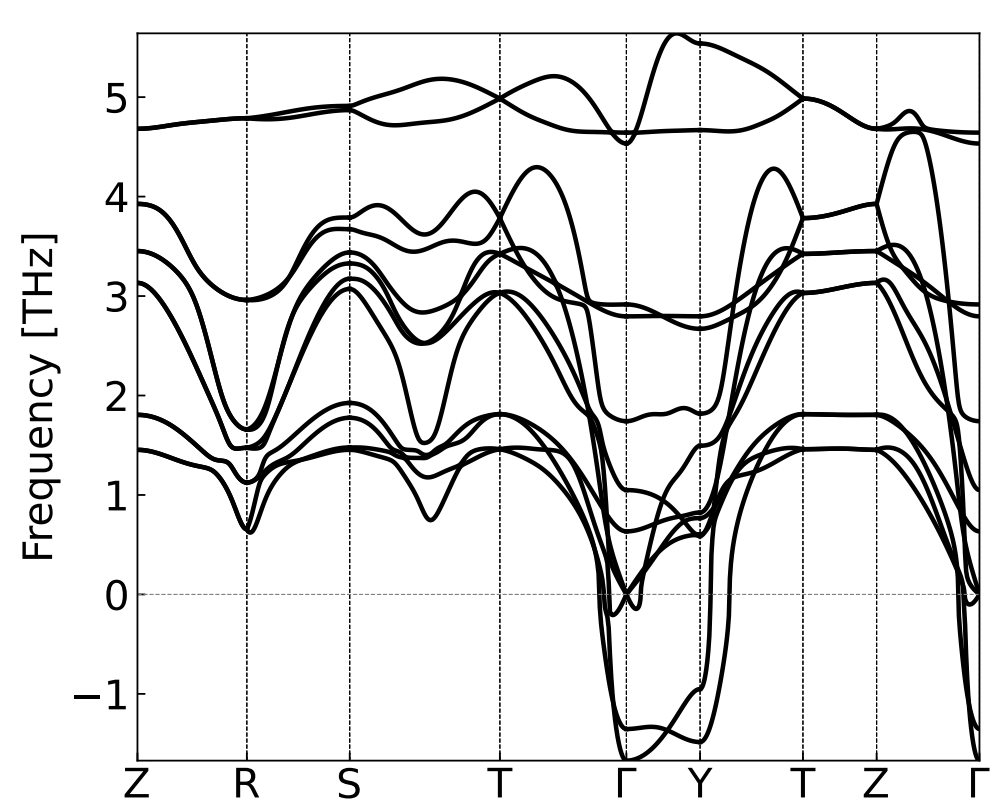

(c)

Figure S4: **The apparent imaginary R-mode in $Cmcm$ SnSe is identified as a Fourier-interpolation artifact.** (a) Comparison of the full phonon band structure of $Cmcm$ SnSe between DFT (black) and the Equitrain model (blue). (b) shows the harmonic potential energy surface for displacements along the imaginary R-mode. Black points denote the calculated DFT energies and the colored surface represents the harmonic fit. (c) DFT calculation with the different supercell matrix S_2.

3. As in scenario 2, DFT exhibits imaginary modes due to interpolation errors. However, the ML model captures an anharmonic potential and relaxes to the same primitive unit cell with same space group, differing only by slightly adjusted lattice parameters. It should be noted here that the energy potential is not correctly represented by the ML model, as a minimally displaced structure is found along the anharmonic direction.

4. Both DFT and ML models exhibit imaginary modes, but the ML model follows an incorrect transition pathway and relaxes into a different space group than obtained

from DFT. An example for this is the transition pathway of K_3Sb in the Multihead Model, which was discussed in the main part section "Imaginary mode prediction and phase transition".

For each material, the set of final phases, S_{ML}, is compared to the set of DFT reference phases, S_{DFT}, as follows: First, the initial phase is removed from both sets of final phases, because it does not represent a new displacive phase and typically corresponds to either scenario 2 or scenario 3. Thus, for an arbitrary phase transition pathway $A \rightarrow B \rightarrow C$, the initial phase A is excluded from both sets S_{ML} and S_{DFT}.

$$S_{ML} \leftarrow S_{ML} \setminus \{A\} \tag{S15}$$

$$S_{DFT} \leftarrow S_{DFT} \setminus \{A\} \tag{S16}$$

The remaining phases are classified as follows:

- $P \in S_{ML}$ is labeled as true positive, if $P \in S_{DFT}$.
- $P \in S_{ML}$ is labeled as false positive if $P \notin S_{DFT}$.
- $P \in S_{DFT}$ is labeled as false negative, if $P \notin S_{ML}$.

The following tables (S2, S3, S4, S5, S6, S7) list all imaginary modes and their corresponding phase-transition pathways for the models specified in the respective table titles. "imag" denotes the total number of imaginary modes found at band minima or high-symmetry q-points. Due to the restriction of a maximum of 1000 atoms per commensurate supercell, only those phase transitions that could be explicitly calculated are shown in the table. Empty entries indicate the absence of imaginary modes. Entries in the DFT Table S2 labeled as "single minimum" indicate the presence of a single (harmonic) minimum as described in scenario 2. "Initial sym." and "#Atoms" denote the space group symmetry and number of atoms in the primitive unit cell of the initial structure, respectively.
"displ. Sym." and "final Sym." refer to the crystal symmetry of the displaced structure at the energy minimum and of the final structure after full geometric optimization, respectively. "ΔE/Atom" denotes the energy difference between the initial and final structure.

Table S2: DFT

material	imag.	q-point	initial sym.	#atoms	displ. sym.	final sym.	#atoms	ΔE/atom [eV]
SnSe (mp-691)	1	0.15/0/0	Pnma	2	Pnma	Pnma	2	0.00011886
SnTe (mp-1883)	2	0/0/0	Fm$\bar{3}$m	2	Cm	Cm	2	0.003926925
		0.5/0/0.5	Fm$\bar{3}$m		P4/nmm	P4/nmm	4	0.000893576
Sb_2Se_3 (mp-2160)	4	0/0/0	Pnma	20	$P2_12_12_1$	$P2_12_12_1$	20	0.000234258
		0/0/0.35			single minimum			
		0.5/0/0	Pnma		$P2_1/c$	$P2_1/c$	40	0.000332583
SnSe (mp-2168)	7	-0.5/0.5/0	Cmcm	4	Pnma	Pnma	8	0.006528244
					Pnma	Pnma	8	0.000847502
		0/0/0			$Cmc2_1$	$Cmc2_1$	4	0.006847633
					Ama2	Ama2	4	0.002075974
		0/0.5/0.5			single minimum			
		0.25/0.25/0.5			Cmcm	Cmcm	4	0.009638485
GeTe (mp-2612)	5	0/0/0	Fm$\bar{3}$m	2	R3m	R3m	2	0.01955798
		0.5/0/0.5	Fm$\bar{3}$m		Pmmn	Pmmn	4	0.003247811
			Fm$\bar{3}$m		P4/nmm	P4/nmm	4	0.000450387
		0.5/0.25/0.75			single minimum			
		0.375/0.375/0.75			single minimum			
		0.625/0.25/0.625			Fm$\bar{3}$m	Fm$\bar{3}$m	2	0.00001452
K_3Sb (mp-14017)	1	0.333/0.333/0.333	$P6_3$/mmc	8	$P6_3$cm	$P6_3$cm	24	0.00090241
As_2PbS_4 (mp-19941)	12	0/0.5/0	Pnma	28	$P2_1/c$	$P2_1/c$	56	0.020323653
					$P2_1/c$	$P2_1/c$	56	0.013101457
		0/0.5/0.5			$P2_1/c$	$P2_1/c$	56	0.015168392
					$P2_1/c$	$P2_1/c$	56	0.015913025
		0.5/0/0			Pnma	$Pna2_1$	28	0.010950972
		0.5/0/0.5			$P2_1/c$	$P2_1/c$	56	0.011384632
		0.5/0.5/0			P$\bar{1}$	P$\bar{1}$	56	0.019336829
		0.5/0.5/0.5			Cc	Cc	56	0.015905725
Bi_2S_3 (mp-22856)	6	0/0/0	Pnma	20	Pnma	Pnma	20	0.00128557
		0/0.05/0			single minimum			
		0.5/0/0			$P2_1/c$	$P2_1/c$	40	0.000950493
Na_2TlSb (mp-866132)	3	0.5/0/0.5	Fm$\bar{3}$m	4	Pmmn	Pmmn	8	0.007805488

Table S3: MP-0b3

material	imag.	q-point	initial sym.	#atoms	displ. sym.	final sym.	#atoms	ΔE/atom [eV]
SnSe (mp-691)	0							
SnTe (mp-1883)	1	0/0/0	$Fm\bar{3}m$	2	P1	R3m	2	0.00356861
Sb_2Se_3 (mp-2160)	3	0/0/0	Pnma	20	$P2_12_12_1$	Pnma	40	0.008583621
		0/0.5/0			$P2_1/c$	$P2_1/c$	40	0.00858354
SnSe (mp-2168)	12	-0.5/0.5/0	Cmcm	4	Pnma	Pnma	8	0.001517438
		0/0/0			Ama2	Ama2	4	0.002312192
					$Cmc2_1$	$Cmc2_1$	4	0.000162019
		0/0/0.025			Ama2	Ama2	160	0.001510682
					Cmcm	Cmcm	4	0.0000000079
					Ama2	Ama2	160	0.0000526411
		0/0.005/0			Cmcm	Cmcm	4	0.0000000079
					Cmcm	Cmcm	4	0.0000000079
		0/0.5/0.5			P1	Cm	8	0.000619386
GeTe (mp-2612)	5	0/0/0	$Fm\bar{3}m$	2	Cm	R3m	2	0.019445665
		0.5/0/0.5			P4/nmm	P4/nmm	4	0.00430617
					$P2_1/m$	Pmmn	4	0.00340047
		0.5/0.25/0.75			C2/m	C2/m	8	0.000002035
		0.375/0.375/0.75			Pmma	Pmma	16	0.002372796
		0.625/0.25/0.625			Pmma	Pmma	16	0.002372796
K_3Sb (mp-14017)	0							
As_2PbS_4 (mp-19941)	22	0/0/0	Pnma	28	$P2_1/c$	$P2_1/c$	28	0.006660053
					$P2_1/c$	$P2_1/c$	28	0.013471486
		0/0/0.5			$Pca2_1$	$Pca2_1$	56	0.009556708
		0/0.5/0			$P2_1/c$	$P2_1/c$	56	0.009728751
					$P2_1/c$	$P2_1/c$	56	0.010450992
		0/0.5/0.5			$P2_1/c$	$P2_1/c$	56	0.010735403
					$P2_1/c$	$P2_1/c$	56	0.011743375
		0.5/0/0			$P2_1/c$	$P2_1/c$	56	0.011743375
					$Pna2_1$	$Pna2_1$	56	0.005817168
		0.5/0/0.5			$P2_1/c$	$P2_1/c$	56	0.008533517
					$P2_1/c$	$P2_1/c$	56	0.006087639
		0.5/0.4/0			$P2_1$	$P2_1$	280	0.011595486
		0.5/0.5/0			P1	$P2_1$	56	0.012269718
		0.5/0.5/0.5			P1	$P\bar{1}$	56	0.012249352
Bi_2S_3 (mp-22856)	2	0/0/0	Pnma	20	$P2_12_12_1$	Pnma	20	0.00701234
		0.5/0/0			Pc	$P2_1/c$	40	0.00701227
Na_2TlSb (mp-866132)	3	0.5/0/0.5	$Fm\bar{3}m$	4	$Fm\bar{3}m$	Pmmn	8	0.014958574
		0.5/0.5/0.5			C2/m	C2/m	8	0.003494006
		0.5/0.25/0.75			C2	$I\bar{4}2m$	16	0.006546509
					I4/mmm	I4/mmm	16	0.002032898

Table S4: From-Scratch

material	imag.	q-point	initial sym.	#atoms	displ. sym.	final sym.	#atoms	ΔE/atom [eV]
SnSe (mp-691)	0							
SnTe (mp-1883)	0							
Sb_2Se_3 (mp-2160)	2	0/.025/0	Pnma	20	$Pmn2_1$	$Pmn2_1$	800	0.001253666
SnSe (mp-2168)	3	-0.5/0.5/0	Cmcm	4	Pnma	$Fm\bar{3}m$	2	0.015364941
		0/0.04/0			Pm	P3m1	50	0.014858382
GeTe (mp-2612)	5	0/0/0	$Fm\bar{3}m$	2	P1	R3m	2	0.005260166
		0.5/0/0.5			Pmmn	Pmmn	4	0.006488827
		0.375/0.375/0.75			Pmma	Pmma	16	0.003595268
		0.625/0.25/0.625			Pmma	Pmma	16	0.003595268
K_3Sb (mp-14017)	0							
As_2PbS_4 (mp-19941)	0							
Bi_2S_3 (mp-22856)	3	0/0/0	Pnma	20	$P2_12_12_1$	$P2_12_12_1$	20	0.001173041
		0/0.075/0			$Pmc2_1$	$Pmc2_1$	800	0.000315138
		0.5/0/0			$P2_1/c$	$P2_1/c$	20	0.001213447
Na_2TlSb (mp-866132)	3	0.5/0/0.5	$Fm\bar{3}m$	4	$P2_1/m$	Pmmn	8	0.006111897

Table S5: L^2-TSP

material	imag.	q-point	initial sym.	#atoms	displ. sym.	final sym.	#atoms	ΔE/atom [eV]
SnSe (mp-691)	0							
SnTe (mp-1883)	1	0/0/0	Fm$\overline{3}$m	2	I4mm	I4mm	2	0.000063844
Sb2Se3 (mp-2160)	4	0/0/0	Pnma	20	P$2_12_12_1$	Pnma	20	0.01031492
		0.5/0/0			Pc	Pnma	20	0.010314876
SnSe (mp-2168)	3	-0.5/0.5/0	Cmcm	4	Pnma	Pnma	8	0.014018347
					Pnma	Pnma	8	0.000127048
		0/0/0			Cmc2_1	Cmc2_1	4	0.006666645
					Ama2	Ama2	4	0.003328276
					P2_1/m	P2_1/m	4	0.003164842
GeTe (mp-2612)	2	0/0/0	Fm$\overline{3}$m	2	P1	R3m	2	0.01579701
		0.5/0/0.5			P2_1/m	Pmmn	4	0.000536886
K_3Sb (mp-14017)	1	0.333/0.333/0.333	P6_3/mmc	8	P6_3cm	P6_3cm	24	0.001109341
As_2PbS_4 (mp-19941)	14	0/0.5/0	Pnma	28	P2_1/c	P2_1/c	56	0.018658018
					P2_1/c	P2_1/c	56	0.013633521
		0/0.5/0.5			P2_1/c	P2_1/c	56	0.014360129
					P2_1/c	P2_1/c	56	0.014077855
		0.5/0.5/0			P1	P2_1	56	0.017369918
		0.5/0.5/0.5			P1	Cc	56	0.018978289
Bi_2S_3 (mp-22856)	4	0/0/0	Pnma	20	P$2_12_12_1$	P$2_12_12_1$	20	0.008654072
		0/0.05/0			Pmc2_1	Pmc2_1	400	0.000915012
		0.5/0/0			Pc	P2_1/c	40	0.008653308
Na_2TlSb (mp-866132)	4	0.5/0/0.5	Fm$\overline{3}$m	4	P2_1/m	Pmmn	8	0.007704081

Table S6: Multihead

material	imag.	q-point	initial sym.	#atoms	displ. sym.	final sym.	#atoms	ΔE/atom [eV]
SnSe (mp-691)	0							
SnTe (mp-1883)	1	0/0/0	Fm$\overline{3}$m	2	Cm	Imm2	2	0.000178059
Sb_2Se_3 (mp-2160)	4	0/0/0	Pnma	20	Pmc2_1	Pmc2_1	20	0.006379553
		0/0/0.05			Pm	Pmc2_1	400	0.00611383
					Pm	Pm	400	0.00282019
		0.5/0/0			Pm	Pm	40	0.006378815
SnSe (mp-2168)	5	-0.5/0.5/0	Cmcm	4	Pnma	Pnma	8	0.031494809
					Pnnm	Pnnm	8	0.024060346
		0/0/0			Cmc2_1	Cmc2_1	4	0.012965835
					Ama2	Ama2	4	0.00548828
GeTe (mp-2612)	2	0/0/0	Fm$\overline{3}$m	2	Cm	R3m	2	0.015541084
		0.5/0/.5			P2_1/m	Pmmn	4	0.002914793
					P4/nmm	P4/nmm	4	0.000237267
K_3Sb (mp-14017)	1	0.333/0.333/0.333	P6_3/mmc	8	R-3	R-3	24	0.000121777
As_2PbS_4 (mp-19941)	28	0.5/0.5/0	Pnma		P1	P$\overline{1}$	56	0.024973003
		0.5/0.5/0.5			P1	P$\overline{2}$	56	0.023414041
Bi_2S_3 (mp-22856)	4	0/0/0	Pnma	20	P$2_12_12_1$	Pnma	20	0.011353954
		0.5/0/0			Pc	Pnma	20	4.388049686
Na_2TlSb (mp-866132)	4							

Table S7: Transfer

material	imag.	q-point	initial sym.	#atoms	displ. sym.	final sym.	#atoms	ΔE/atom [eV]
SnSe (mp-691)	0							
SnTe (mp-1883)	0							
Sb_2Se_3 (mp-2160)	4	0/0/0	Pnma	20	$P2_12_12_1$	$P2_12_12_1$	2	0.002272407
		0.5/0/0			Pc	$P2_1/c$	40	0.002273069
		0.375/0/0			Pc	Pc	160	0.001846368
					Pc	Pc	160	0.001826809
SnSe (mp-2168)	5	0/0/0	Cmcm	4	Ama2	Ama2	4	0.004294459
					$Cmc2_1$	$Cmc2_1$	4	0.005555749
		-0.5/0.5/0			Pnma	Pnma	8	0.010656904
		0/0.025/0			Pc	$P2_1/c$	160	0.003972777
					Pm	Pm	160	0.002505208
					Pm	Pm	160	0.002503663
GeTe (mp-2612)	2	0/0/0	$Fm\bar{3}m$	2	P1	R3m	2	0.014341852
		0.5/0/0.5			Pmmn	Pmmn	4	0.000859241
K_3Sb (mp-14017)	0							
As_2PbS_4 (mp-19941)	12	0/0.5/0	Pnma	28	$P2_1/c$	$P2_1/c$	56	0.016478829
					$P2_1/c$	$P2_1/c$	56	0.013351529
		0.5/0.5/0			P1	$P2_1$	56	0.015471764
		0.5/0.5/0.5			P1	$P\bar{1}$	56	0.015489667
		0/0.5/0.5			$P2_1/c$	$P2_1/c$	56	0.01542228
					$P2_1/c$	$P2_1/c$	56	0.013614605
Bi_2S_3 (mp-22856)	4	0/0/0	Pnma	20	$P2_12_12_1$	$P2_12_12_1$	20	0.004766275
		0.5/0/0			Pc	$P2_1/c$	40	0.004766028
Na_2TlSb (mp-866132)		0.5/0.25/0.75	$Fm\bar{3}m$	4	C2	Fddd	16	0.000099165

Comparison of L^2-TSP with LoRA

L^2-TSP and LoRA constrain fine-tuning in different ways. L^2-TSP combines L^2-SP regularization with selective layer fine-tuning, thereby penalizing deviations from the pre-trained parameters while restricting adaptation to a selected subset of the network. In contrast, LoRA constrains the additive parameter update through a low-rank factorization. A LoRA implementation for MACE was introduced by Tompa et al. [10], which supports rank-constrained updates and recommends using zero weight decay.

To compare the two approaches, we trained models using the MACE LoRA implementation with ranks $r \in 4, 16, 64$. Following the recommended setup, the LoRA scaling factor was set to $\alpha = 1$ and the weight decay to $\lambda = 0$. All remaining hyperparameters were kept identical to those used for L^2-TSP. The models were trained on the large-supercell datasets, and the resulting phonon band structures were evaluated against the DFT benchmark. Table S8 reports the median phonon MAE and the corresponding IQR across all 53 materials. For all investigated ranks, the MACE LoRA implementation [10] yields substantially larger phonon errors than L^2-TSP, demonstrating a clear advantage of L^2-TSP over low-rank LoRA for the present phonon-prediction task.

Table S8: Median phonon MAE and IQR in THz of all 53 material models of the L^2-TSP and LoRA approaches with different rank.

	L^2-TSP	LoRA (r=4)	LoRA (r=16)	LoRA (r=64)
large supercell	**0.05 (0.05)**	0.17 (0.13)	0.19 (0.13)	0.18 (0.13)

For all investigated ranks, the low-rank LoRA implementation in MACE yields substantially larger phonon errors than L^2-TSP. Instead, L^2-TSP achieves a median MAE of 0.05 THz, compared with 0.17–0.19 THz for the low-rank models. Furthermore, increasing the rank from $r = 4$ to $r = 64$ does not systematically improve the results, leading to the assumption that the observed difference is not caused solely by the limited rank of the LoRA update. Within the used set of hyperparameters, L^2-SP fine-tuning provides a strong advantage for phonon prediction.

Effect of layer selection in transfer learning and L^2-SP fine-tuning

Layer freezing restricts fine-tuning to a subset of the pre-trained model parameters and can reduce the computational cost of adaptation, particularly for large training datasets [11]. Here, we treat layer freezing as a mechanism for selecting which parts of the network are allowed to adapt rather than as a separate fine-tuning strategy. We therefore systematically investigate the effect of the trainable layer subset for both transfer learning and L^2-SP.

The MACE-MP-0b3 architecture considered here contains six layers, executed in the following order: (1) node embedding, (2) interaction 0, (3) product 0, (4) interaction 1, (5) product 1, and (6) readout. We varied contiguous subsets of active layers while keeping all remaining layers frozen. The same layer restrictions were applied to both L^2-SP and transfer learning in order to compare their sensitivity to the choice of trainable parameters.

Table S9 reports the resulting median phonon MAEs and corresponding IQRs. Apart from the regularization term specific to each approach, the training settings were kept identical.

For L^2-SP, the lowest phonon error is obtained when only the first two layers, corresponding to the node embedding and the first interaction layer, are adapted. This configuration yields a median phonon MAE of 0.05 THz and is therefore used as the targeted L^2-SP (L^2-TSP) configuration throughout the main analysis. The L^2-SP results depend strongly on the selected trainable layers: activating additional layers beyond the first three leads to a pronounced increase in phonon error.

Transfer learning is comparatively robust with respect to layer selection and reaches similar phonon errors for several different subsets of trainable layers. In particular, transfer learning with layers 1–2 active yields the same median phonon MAE of 0.05 THz as the selected L^2-TSP configuration. Thus, for harmonic phonon accuracy, the present comparison does not show a clear advantage of L^2-SP regularization over transfer learning with the same layer restriction. Among the transfer-learning configurations, layers 3–6 yield one of the lowest median phonon MAEs, 0.05 THz, together with the smallest IQR of 0.04 THz. We therefore carry this configuration forward to additional tests of dynamical stability and phase recovery to assess whether comparable harmonic phonon accuracy also translates into comparable behavior beyond the near-equilibrium training configurations.

As an additional control, transfer learning was repeated with conventional weight decay of $wd = 10$, equal in magnitude to the coefficient applied to the residual parameters in L^2-SP. This substantially increases the phonon errors. Conventional weight decay penalizes the magnitude of the model parameters and drives them toward zero, whereas L^2-SP penalizes their displacement from the pre-trained parameters. Applying the same numerical coefficient therefore corresponds to fundamentally different regularization objectives.

Table S9: Median phonon MAE and IQR in THz of all 53 material models of the L^2-TSP fine-tuning and transfer learning, as implemented in Equitrain, with different subsets of active layers. All remaining layers were frozen during training.

active fine-tuned layers	L^2-TSP	Transfer Learning	Transfer Learning (wd=10)
1	0.20 (0.16)	0.08 (0.06)	**0.28 (0.17)**
1-2	**0.05 (0.05)**	0.05 (0.05)	0.43 (0.30)
1-3	0.06 (0.09)	0.05 (0.05)	0.47 (0.46)
1-4	0.46 (0.85)	0.06 (0.05)	1.28 (1.64)
1-5	1.43 (1.56)	0.06 (0.05)	1.49 (1.56)
6	0.20 (0.15)	0.09 (0.07)	0.38 (0.29)
5-6	0.10 (0.08)	0.06 (0.04)	0.38 (0.39)
4-6	0.06 (0.05)	0.06 (0.04)	1.42 (1.07)
3-6	0.18 (0.28)	**0.05 (0.04)**	1.54 (1.15)
2-6	0.06 (0.05)	0.07 (0.05)	1.89 (1.93)

Since transfer learning with active layers 3–6, while all remaining layers are frozen, yields harmonic phonon errors comparable to those of L^2-TSP, we further evaluate this configuration for dynamical instabilities and phase recovery. The results are summarized in Table S10

together with the corresponding results for the foundation model, conventional transfer learning, multi-head fine-tuning, and L^2-TSP.

Transfer learning with active layers 3–6 reproduces the dynamical-stability classification of L^2-TSP exactly, with all 44 DFT-stable materials classified correctly and eight of the nine unstable materials identified, corresponding to an imaginary-mode precision of 100% and recall of 89%. However, this agreement does not fully carry over to the recovery of the associated phases. The selectively fine-tuned transfer model achieves a precision of 61%, a recall of 58%, and an F1 score of 0.60. This improves upon conventional transfer learning ($F_1 = 0.56$), but remains below both the foundation model MP-0b3 ($F_1 = 0.63$) and L^2-TSP ($F_1 = 0.66$). Thus, comparable harmonic phonon accuracy and even identical dynamical-stability classification do not necessarily imply comparable performance in recovering the phases associated with unstable phonon modes.

Table S10: Comparison of dynamical-stability classification and phase-recovery performance. Transfer (layers 3–6) denotes transfer learning in which only layers 3–6 are fine-tuned and all remaining layers are frozen. Precision and recall for dynamical stability are calculated with respect to imaginary modes. For phase recovery, precision and recall are calculated separately for each material and averaged as described in the main text.

	MP-0b3	Transfer	Transfer (layers 3–6)	Multihead	L^2-TSP
Dynamical-stability classification					
True stable	42	43	**44**	42	**44**
False stable	2	1	**0**	2	**0**
False unstable	2	3	**1**	**1**	**1**
True unstable	7	6	**8**	**8**	**8**
Imaginary-mode precision	78%	86%	**100%**	80%	**100%**
Imaginary-mode recall	78%	67%	**89%**	**89%**	**89%**
Recovery of DFT reference phases					
Precision	60%	54%	61%	38%	**68%**
Recall	**66%**	58%	58%	50%	65%
F1 score	0.63	0.56	0.60	0.43	**0.66**

References

[1] Ilyes Batatia et al. “A foundation model for atomistic materials chemistry”. In: *The Journal of Chemical Physics* 163.18 (2025).

[2] Ilyes Batatia et al. “Cross learning between electronic structure theories for unifying molecular, surface, and inorganic crystal foundation force fields”. In: *arXiv preprint arXiv:2510.25380* (2025).

[3] Huiju Lee et al. “Accelerating high-throughput phonon calculations via machine learning universal potentials”. In: *Materials Today Physics* 53 (2025), p. 101688.

[4] Janosh Riebesell et al. *Pymatviz: visualization toolkit for materials informatics*. Version 0.8.2. 10.5281/zenodo.7486816 - https://github.com/janosh/pymatviz. Oct. 1, 2022. DOI: `10.5281/zenodo.7486816`. URL: `https://github.com/janosh/pymatviz` (visited on 01/01/2023).

[5] Atsushi Togo. “First-principles Phonon Calculations with Phonopy and Phono3py”. In: *J. Phys. Soc. Jpn.* 92.1 (2023), p. 012001. DOI: `10.7566/JPSJ.92.012001`.

[6] Glen A Slack. “Nonmetallic crystals with high thermal conductivity”. In: *Journal of Physics and Chemistry of Solids* 34.2 (1973), pp. 321–335.

[7] Tiantian Jia, Gang Chen, and Yongsheng Zhang. “Lattice thermal conductivity evaluated using elastic properties”. In: *Physical Review B* 95.15 (2017), p. 155206.

[8] Alex M Ganose et al. “Atomate2: Modular workflows for materials science”. In: *Digital Discovery* (2025).

[9] M Hebbache and M Zemzemi. “Ab initio study of high-pressure behavior of a low compressibility metal and a hard material: Osmium and diamond”. In: *Physical Review B—Condensed Matter and Materials Physics* 70.22 (2004), p. 224107.

[10] Tamás Lajos Tompa et al. “Fine-tuning MLIP foundation models: strategies for accuracy and transferability”. In: *arXiv preprint arXiv:2606.12704* (2026).

[11] Mariia Radova et al. “Fine-tuning foundation models of materials interatomic potentials with frozen transfer learning”. In: *npj Computational Materials* 11.1 (2025), p. 237.
